# Diamond magnetometer enhanced by ferrite flux concentrators

**DOI:** 10.1103/physrevresearch.2.023394

**Published:** 2020-06-24

**Authors:** Ilja Fescenko, Andrey Jarmola, Igor Savukov, Pauli Kehayias, Janis Smits, Joshua Damron, Nathaniel Ristoff, Nazanin Mosavian, Victor M. Acosta

**Affiliations:** 1 Center for High Technology Materials and Department of Physics and Astronomy, University of New Mexico, Albuquerque, New Mexico 87106, USA; 2 ODMR Technologies Inc., El Cerrito, California 94530, USA; 3 Department of Physics, University of California, Berkeley, California 94720, USA; 4 Los Alamos National Laboratory, Los Alamos, New Mexico 87545, USA; 5 Sandia National Laboratory, Albuquerque, New Mexico 87185, USA; 6 Laser Center of the University of Latvia, Riga LV-1004, Latvia

## Abstract

Magnetometers based on nitrogen-vacancy (NV) centers in diamond are promising room-temperature, solid-state sensors. However, their reported sensitivity to magnetic fields at low frequencies (≾1 kHz) is presently ≿10 pT s^1/2^, precluding potential applications in medical imaging, geoscience, and navigation. Here we show that high-permeability magnetic flux concentrators, which collect magnetic flux from a larger area and concentrate it into the diamond sensor, can be used to improve the sensitivity of diamond magnetometers. By inserting an NV-doped diamond membrane between two ferrite cones in a bowtie configuration, we realize a ~250-fold increase of the magnetic field amplitude within the diamond. We demonstrate a sensitivity of ~0.9 pT s^1/2^ to magnetic fields in the frequency range between 10 and 1000 Hz. This is accomplished using a dual-resonance modulation technique to suppress the effect of thermal shifts of the NV spin levels. The magnetometer uses 200 mW of laser power and 20 mW of microwave power. This work introduces a new degree of freedom for the design of diamond sensors by using structured magnetic materials to manipulate magnetic fields.

## I. INTRODUCTION

Quantum sensors based on nitrogen-vacancy (NV) centers in diamond have emerged as a powerful platform for detecting magnetic fields across a range of length scales [1]. At the few-nanometer scale, single NV centers have been used to detect magnetic phenomena in condensed-matter [2,3] and biological [4,5] samples. At the scale of a few hundred nanometers, diamond magnetic microscopes have been used to image biomagnetism in various systems, including magnetically labeled biomolecules [6] and cells [7,8] and intrinsically magnetic biocrystals [9,10]. At the micrometer scale, diamond magnetometers have detected the magnetic fields produced by neurons [11], integrated circuits [12,13], and the nuclear magnetic resonance of fluids [14,15].

Diamond magnetometers with larger active volumes are expected to offer the highest sensitivity [16]. However, in order to be competitive with existing technologies, they must overcome several technical drawbacks, including high laser-power requirements and poor sensitivity at low frequencies. The most sensitive diamond magnetometer reported to date featured a projected sensitivity of ~0.9 pT s^1/2^ using 400 mW of laser power [17]. However this magnetometer used a Hahn-echo pulse sequence which limited the bandwidth to a narrow range around 20 kHz. For broadband, low-frequency operation, the highest sensitivity reported to date is ~15 pT s^1/2^ in the 80–2000 Hz range, using ≿3 W of laser power [11]. A diamond magnetometer based on infrared absorption detection realized a sensitivity of ~30 pT s^1/2^ at 10–500 Hz, using 0.5 W of laser power [18].

To understand the interplay between sensitivity and laser power, we consider a diamond magnetometer based on continuous-wave, fluorescence-based optically detected magnetic resonance (ODMR). The sensitivity is fundamentally limited by photoelectron shot noise as

(1) $$\eta_{\text{psn}}\approx \frac{\Gamma }{\gamma_{\text{nv}}C\sqrt{\xi P_{\text{opt}}/E_{\text{ph}}}},$$

where *γ*_nv_ = 28 GHz/T is the NV gyromagnetic ratio, Γ is the ODMR full-width-at-half-maximum linewidth, and *C* is the ODMR amplitude’s fractional contrast. The factor *ξP*_opt_/*E*_ph_ constitutes the photoelectron detection rate, where *P*_opt_ is the optical excitation power, *ξ* is the fraction of excitation photons converted to fluorescence photoelectrons, and *E*_ph_ = 3.7 × 10^−19^ J is the excitation photon energy (532 nm). To set an optimistic bound on *η*_psn_, we insert the best reported values (*ξ* = 0.08 [17], Γ/*C* = 1 MHz/0.04 [11]) into Eq. (1) to obtain $\eta_{\text{psn}}\approx 2{\text{pT s}}^{1/2}{\text{W}}^{1/2}P_{\text{opt}}^{-1/2}$. Even in this ideal case (Appendix M), ~4 W of optical power is needed to realize a sensitivity of 1 pT s^1/2^, and further improvements become impractical.

The need for such a high laser power presents challenges for thermal management and has implications for the overall sensor size, weight and cost. Applications which call for sub-picotesla sensitivity, such as magnetocardiography (MCG) [19], magnetoencephalography (MEG) [20], and long-range magnetic anomaly detection [21,22], may require alternative approaches to improve sensitivity. Avenues currently being pursued often focus on reducing the ratio Γ/*C* [16]. Approaches to reduce include lowering ^13^C spin density and mitigating strain and electric-field inhomogeneity [23,24], increasing the nitrogen-to-NV^−^ conversion yield [25–27], and designing techniques to decouple NV centers from paramagnetic spins [24,28]. Methods to increase *C* include using preferentially aligned NV centers [29,30], detecting infrared absorption [18,31], and detecting signatures of photo-ionization [32–34].

In this paper, we report a complementary approach to improve the sensitivity of diamond magnetometers. Our approach uses magnetic flux concentrators to amplify the external magnetic field amplitude by a factor of ~250 within the diamond sensor. Using a dual-resonance magnetometry technique to suppress the effect of thermal shifts of the NV spin levels, we realize a sensitivity of ~0.9 pT s^1/2^ in the 10–1000 Hz range, using a laser power of 200 mW. We show that, with further improvements, a magnetic noise floor of ~0.02 pT s^1/2^ at 1000 Hz is possible before ferrite thermal magnetization noise limits the sensitivity.

## II. EXPERIMENTAL DESIGN

Magnetic flux concentrators have previously been used to improve the sensitivity of magnetometers based on the Hall effect [35], magnetoresistance [36], magnetic tunnel junctions [37], superconducting quantum interference devices (SQUIDs) [38], and alkali spin precession [39]. Typically, the magnetometer is positioned in the gap between a pair of ferromagnetic structures which collect magnetic flux from a larger area and concentrate it into the gap. The fractional increase in magnetic field amplitude due to the flux concentrators, *ϵ*, is a function of their geometry, gap width, and relative permeability (*μ*_*r*_). Ideally, the concentrators are formed from a soft magnetic material with low remanence, high *μ*_*r*_, low relative loss factor [39], and constant susceptibility over a broad range of magnetic field amplitudes and frequencies. The improvement in sensitivity is generally accompanied by a reduction in spatial resolution, as the total magnetometer size is larger (Appendix B). Diamond sensors usually have sub-mm dimensions, whereas the flux concentrators used here have dimensions of ~10 mm. Thus our device is best suited for applications that require a spatial resolution ≿10 mm, such as MCG, MEG, and magnetic anomaly detection.

**FIG. 1.**
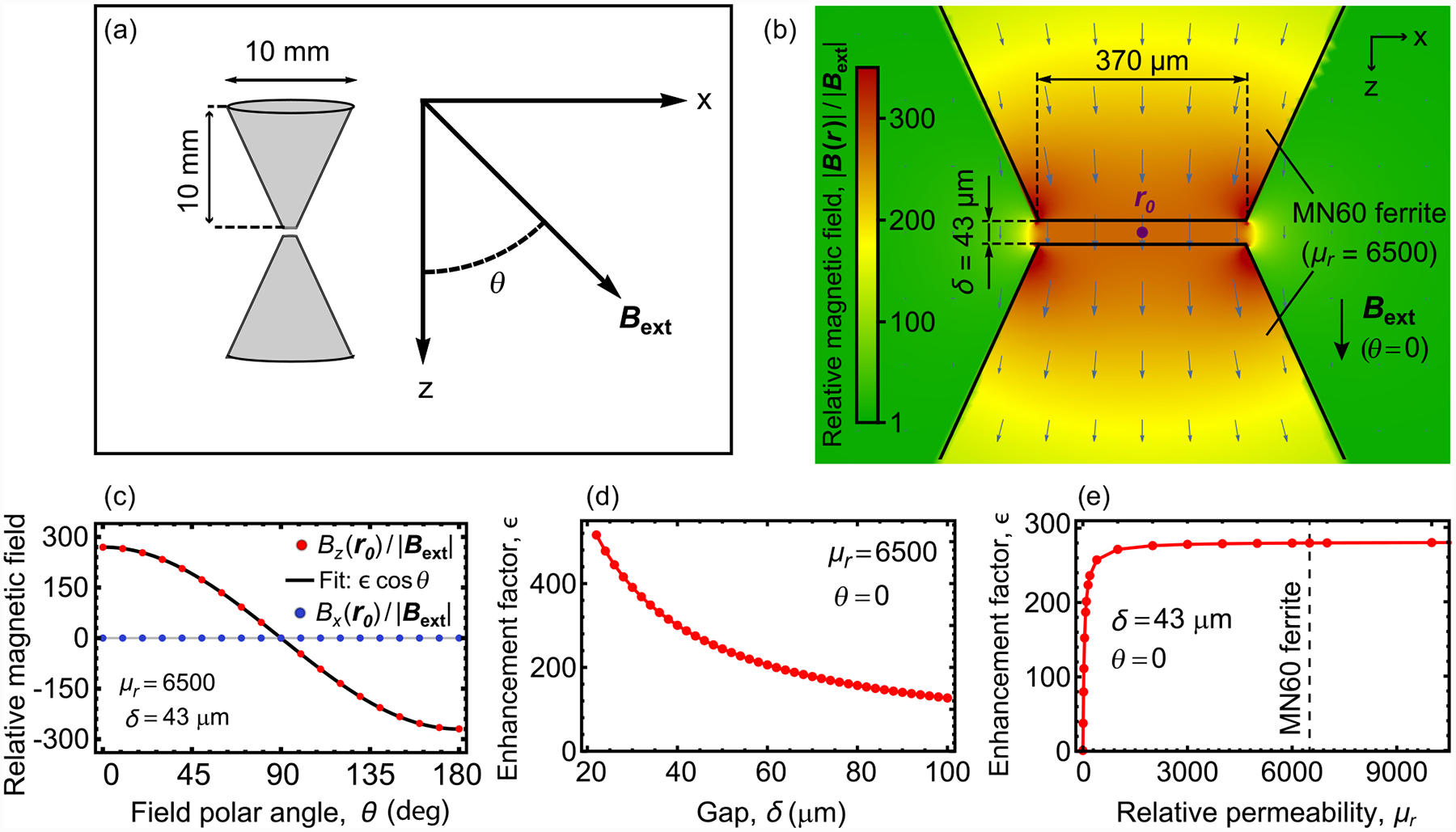
Simulations of magnetic flux concentrators: (a) Model geometry. Two identical solid cones, configured in a bowtie geometry, are placed in an external magnetic field, ***B***_**ext**_. (b) Simulated *x*-*z* plane cut of the relative magnetic field amplitude, |***B***(***r***)|/|***B***_**ext**_|, for cones with relative permeability *μ*_*r*_ = 6500 and a tip gap of *δ* = 43 μm, upon application of ***B***_**ext**_ at *θ* = 0. Arrows indicate the direction and magnitude of ***B***(***r***). The point at the geometric center is labeled ***r***_**0**_. (c) Vector components of the relative magnetic field amplitude at ***r***_**0**_ as a function of *θ*, for cones with *μ*_*r*_ = 6500 and *δ* = 43 μm. The relative axial magnetic field amplitude is fit to the function *B*_*z*_(***r*0**)/|***B***_**ext**_| = *ϵ* cos *θ*, where in this case *ϵ* = 280. (d) Enhancement factor as a function of *δ* for cones with *μ*_*r*_ = 6500. (e) Enhancement factor as a function of *μ*_*r*_ for *δ* = 43 μm.

The optimal flux concentrator geometry depends on a number of factors, which include the sensor dimensions and target application [35,36,38–40]. Here we consider a pair of identical cones (height: 10 mm, base diameter: 10 mm), with ~370-μm diameter flat tips, arranged in a bowtie configuration [Fig. 1(a)]. A static magnetic field, ***B***_**ext**_, is applied at an angle *θ* from the cone symmetry axis ($\hat{z}$) and the resulting magnetic field, ***B*(*r*)**, is simulated using finite-element magnetostatic methods. Figure 1(b) shows a plane cut of the relative magnetic field amplitude, |***B***(***r***)|/|***B***_**ext**_|, for cones with *μ*_*r*_ = 6500 and a tip gap of *δ* = 43 μm, upon application of ***B***_**ext**_ at *θ* = 0. Throughout the gap (Appendix B), ***B***(***r***) is aligned along $\hat{z}$ with a uniform relative magnetic field |***B***(***r***)|/*|****B***_**ext**_| ≈ 280.

Figure 1(c) shows the vector components of the relative magnetic field at the center of the bowtie geometry (***r*** = ***r***_**0**_) as a function of *θ*. The relative axial magnetic field is well described by *B*_*z*_(***r***_**0**_)/|***B***_**ext**_| ≈ *ϵ* cos *θ*, where *ϵ* is the enhancement factor (in this simulation *ϵ* = 280). On the other hand, the relative transverse magnetic field, *B*_*x*_(***r***_**0**_)/|***B***_**ext**_|, is less than 0.1 for all values of *θ*. Thus, the structure acts as a filter for the axial component of external magnetic fields, producing a uniform field throughout the gap of

(2) $$B_{\text{gap}}\approx \epsilon \left|B_{\text{ext}}\right|\text{cos}\theta \hat{z}.$$

For the remainder of the paper, we consider only external magnetic fields applied along $\hat{z}(\theta =0)$ and describe ***B***_**gap**_ according to Eq. (2).

Figure 1(d) shows simulation results of the enhancement factor as a function of gap length for cones with *μ*_*r*_ = 6500. For *δ* in the 20–100 μm range, *ϵ* varies from 560 to 120, indicating that large enhancement factors are possible for typical diamond membrane thicknesses. Figure 1(e) is a plot of the simulated *ϵ* as a function of *μ*_*r*_ for *δ* = 43 μm. For *μ*_*r*_ ≿ 500 the enhancement factor is relatively constant at *ϵ* ≈ 280. This indicates that a wide range of magnetic materials can be used for flux concentration and minor variations in *μ*_*r*_ (due, for example, to temperature variation; Appendix S) have a negligible impact on ***B***_**gap**_.

**FIG. 2.**
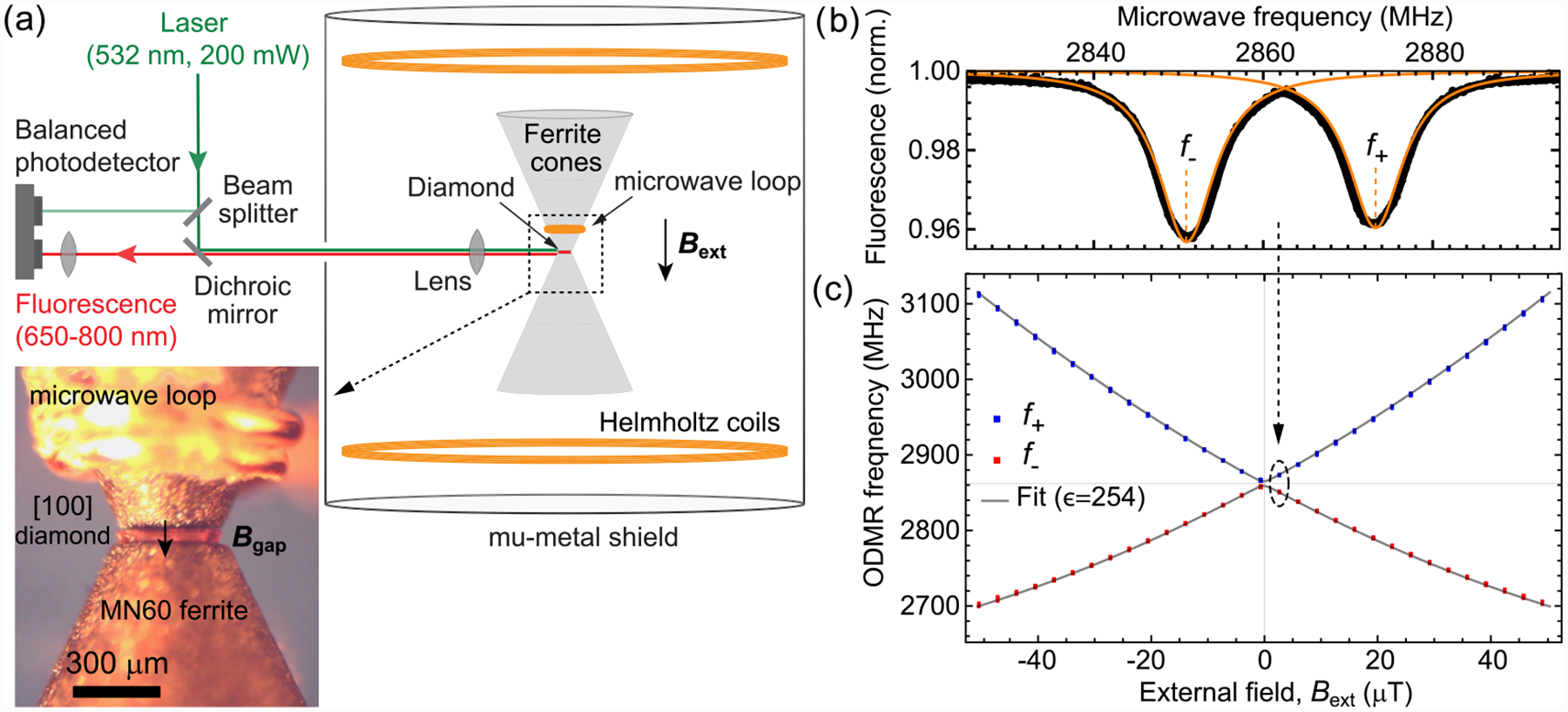
Experimental setup and enhancement measurement: (a) Schematic of the experimental setup. Inset: photograph of the diamond membrane in the gap between ferrite cones. (b) Optically detected magnetic resonance (ODMR) spectrum obtained at *B*_ext_ = 2.62 μT. Two peaks are present, with central frequencies *f*_±_ extracted from Lorentzian fits. (c) Measured ODMR frequencies as a function of *B*_ext_. Error bars are smaller than the plot markers. The gray solid lines are a fit using the NV spin Hamiltonian (Appendix A), assuming *B*_gap_ = *ϵB*_ext_, with *ϵ* = 254.

We elected to use MN60 ferrite (*μ*_*r*_ ≈ 6500) as the experimental concentrator material, owing to its low thermal magnetic noise [39,41]. The ferrite cones were machined to have approximately the same dimensions as simulated in Fig. 1. Figure 2(a) depicts the experimental setup. An NV-doped diamond membrane with [100] faces is positioned in the gap between the ferrite cones. The membrane was formed from a commercially available, type Ib diamond grown by high-pressure high-temperature (HPHT) synthesis. The diamond had been irradiated with 2-MeV electrons at a dose of ~10^19^ cm^−2^. It was subsequently annealed in a vacuum furnace at 800–1100 °C [9] and mechanically polished and cut into a membrane of dimensions ~300 × 300 × 43 μm^3^.

Approximately 200 mW of light from a 532 nm laser is focused by a 0.79 NA lens to a ~40 μm diameter beam that traverses the diamond membrane parallel to its faces. The same lens is used to collect NV fluorescence, which is then refocused onto one of the channels of a balanced photodetector, producing ~1.2 mA of photocurrent. A small portion of laser light is picked off from the excitation path and directed to the other photodetector channel for balanced detection. Microwaves are delivered by a two-turn copper loop wound around one of the ferrite cones. The ferrite cones provide a ≿ twofold enhancement in the microwave magnetic field amplitude within the diamond (Appendix I). All measurements were performed using ≾20 mW of microwave power.

The ferrite-diamond assembly is positioned at the center of a pair of Helmholtz coils (radius: 38 mm), which produce a homogenous magnetic field parallel to the cone axis of amplitude *B*_ext_. The coils’ current response was calibrated using three different magnetometers (Appendix J). A 1.5-mm-thick cylindrical mu-metal shield (diameter: 150 mm, height: 150 mm) surrounds the Helmholtz coils, providing a shielding factor of ~100.

To measure the enhancement factor, we recorded the NV ODMR spectrum as a function of *B*_ext_. Figure 2(b) shows a typical ODMR spectrum acquired at *B*_ext_ = 2.62 μT. Two peaks are present, with central frequencies *f*_±_. These frequencies correspond to NV electron-spin transitions between the *m*_*s*_ = 0 and *m*_*s*_ = ±1 magnetic sublevels (Appendix A). For magnetic field amplitudes within the diamond in the range 0.5 mT ≾ *ϵ B*_ext_ ≾ 5 mT, the transition frequencies may be approximated as

(3) $$f_{\pm }\approx D(\Delta T)\pm \gamma_{\text{nv}}\epsilon B_{\text{ext}}/\sqrt{3},$$

where, in our experiments (Appendix D), *D*(Δ*T*) ≈ 2862 MHz + *χ*Δ*T* is the axial zero-field splitting parameter which shifts with changes in temperature, Δ*T*, as *χ* ≈ −0.1 MHz/K [42]. The $1/\sqrt{3}$ factor in Eq. (3) comes from projecting ***B***_**gap**_ onto the four NV axes which are all aligned at 55° with respect to the cone axis.

Figure 2(c) plots the fitted *f*_±_ values as a function of *B*_ext_. These data were obtained by scanning *B*_ext_ back and forth between ±50 μT two times. For a given *B*_ext_, the extracted *f*_±_ are nearly identical regardless of scan history, indicating negligible hysteresis (Appendix L). The data were fit according to the NV spin Hamiltonian (Appendix A), which reveals an experimental enhancement factor of *ϵ* = 254 ± 19. The uncertainty in *ϵ* is primarily due to uncertainty in the *B*_ext_ current calibration (Appendix J). The experimental enhancement factor is ~10% smaller than the one simulated in Fig. 1(b). This could be explained by a ~4 μm increase in *δ* due to adhesive between the diamond and ferrite tips (Appendix C).

Having established that the ferrite cones provide a ~250-fold field enhancement, we now turn to methods of using the device for sensitive magnetometry. A common approach in diamond magnetometry [43,44] is to modulate the microwave frequency about one of the ODMR resonances and demodulate the resulting fluorescence signal using a lock-in amplifier (Appendix F). We call this method “single-resonance” magnetometry, as each resonance frequency is measured independently. For example, to measure *f*_+_, the microwave frequency is varied as $F(t)\approx f_{+}+f_{d}\text{cos}\left(2\pi f_{\text{mod}}t\right)$, where *f*_*d*_ is the modulation depth and *f*_mod_ is the modulation frequency. The lock-in amplifier demodulates the photodetector signal using a reference signal proportional to cos (2*π f*_mod_*t*). The resulting lock-in output is proportional to variations in *f*_+_.

However, a single ODMR resonance can shift due to changes in *temperature* in addition to magnetic field; see Eq. (3). To isolate the shifts due only to changes in magnetic field, the difference frequency (*f*_+_ − *f*_−_) must be determined. Previous works accomplished this by measuring both resonances either sequentially [45] or simultaneously by multiplexing modulation frequencies [46,47]. The magnetic field was then inferred by measuring *f*_+_ and *f*_−_ independently and calculating the difference.

**FIG. 3.**
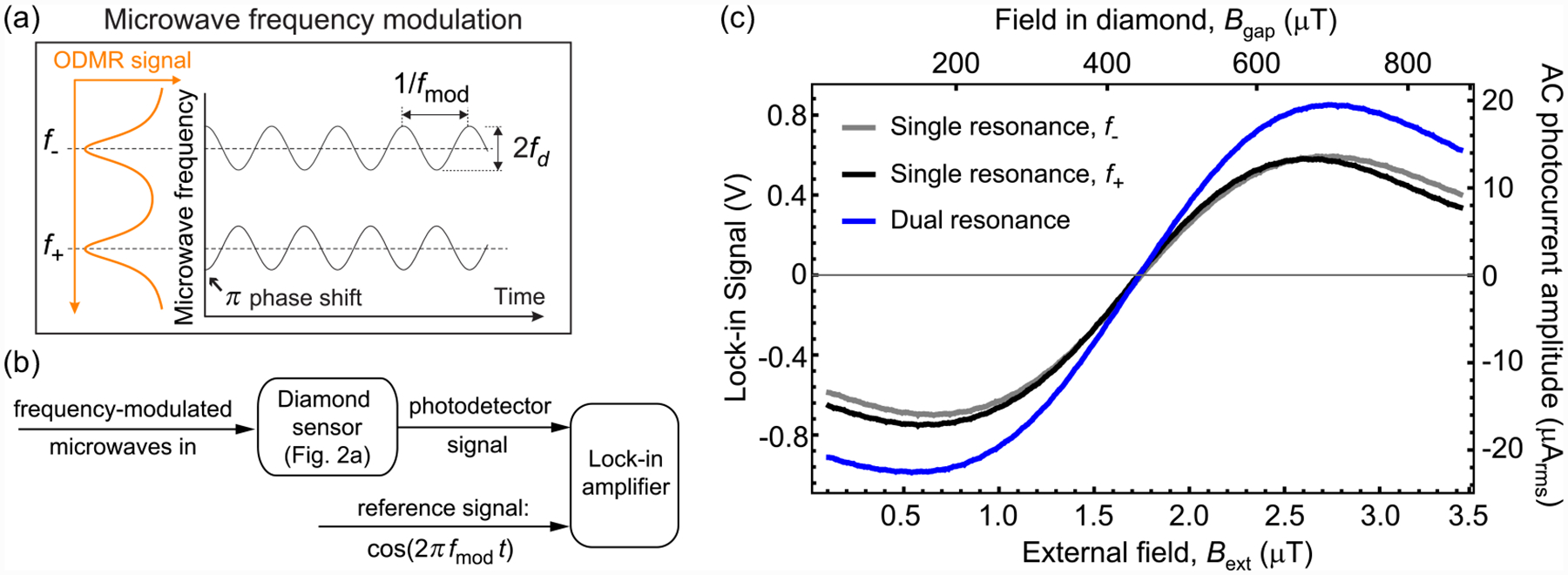
Dual-resonance magnetometry concept: (a) Microwave frequency modulation used for dual-resonance magnetometry. (b) Schematic of the lock-in technique. Both microwave signals depicted in (a) are combined and delivered through the microwave loop. NV fluorescence is continuously excited and its time-varying intensity is recorded by the balanced photodetector. This signal is then fed to a lock-in amplifier and demodulated by the reference signal. (c) Lock-in signal as a function of *B*_ext_ for both single-resonance and dual-resonance modulation protocols. The microwave frequencies were centered about the *f*_±_ values measured by ODMR spectroscopy at *B*_ext_ = 1.73 μT. In all cases, *f*_mod_ = 15 kHz, and the lock-in uses a 12 dB/octave low-pass filter with a 100 μs time constant. For the *f*_−_ scan, the lock-in reference signal had a *π* phase shift relative to the modulation function. The right vertical axis converts the lock-in signal to the amplitude of photocurrent oscillations at *f*_mod_, which is used to estimate the photoelectron-shot-noise-limited sensitivity, Appendix M.

Here, we use an alternative “dual-resonance” approach, which extracts the magnetic field amplitude directly from a single lock-in measurement (Appendix F). Two microwave signal frequencies, centered about *f*_±_, are modulated to provide time-varying frequencies, $F_{\pm }(t)\approx f_{\pm }\pm f_{d}\text{cos}\left(2\pi f_{\text{mod}}t\right)$. In other words, each tone is modulated with the same modulation frequency and depth, but with a relative *π* phase shift [Fig. 3(a)]. The photodetector signal is then demodulated by the lock-in amplifier using a reference signal proportional to cos (2*π f*_mod_*t*) [Fig. 3(b)]. In this way, the lock-in output is proportional to (*f*_+_ − *f*_−_) and is unaffected by thermal shifts of *D*(Δ*T*). Furthermore, the dual-resonance lock-in signal’s response to magnetic fields is larger than in the single-resonance case. Figure 3(c) shows the experimental lock-in signal as a function of *B*_ext_ for dual-resonance modulation and both of the *f*_±_ single-resonance modulation protocols. The slope for dual-resonance modulation is ~1.3 times larger than that of single-resonance modulation. This is close to the expected increase of 4/3 (Appendix G).

## III. RESULTS

**FIG. 4.**
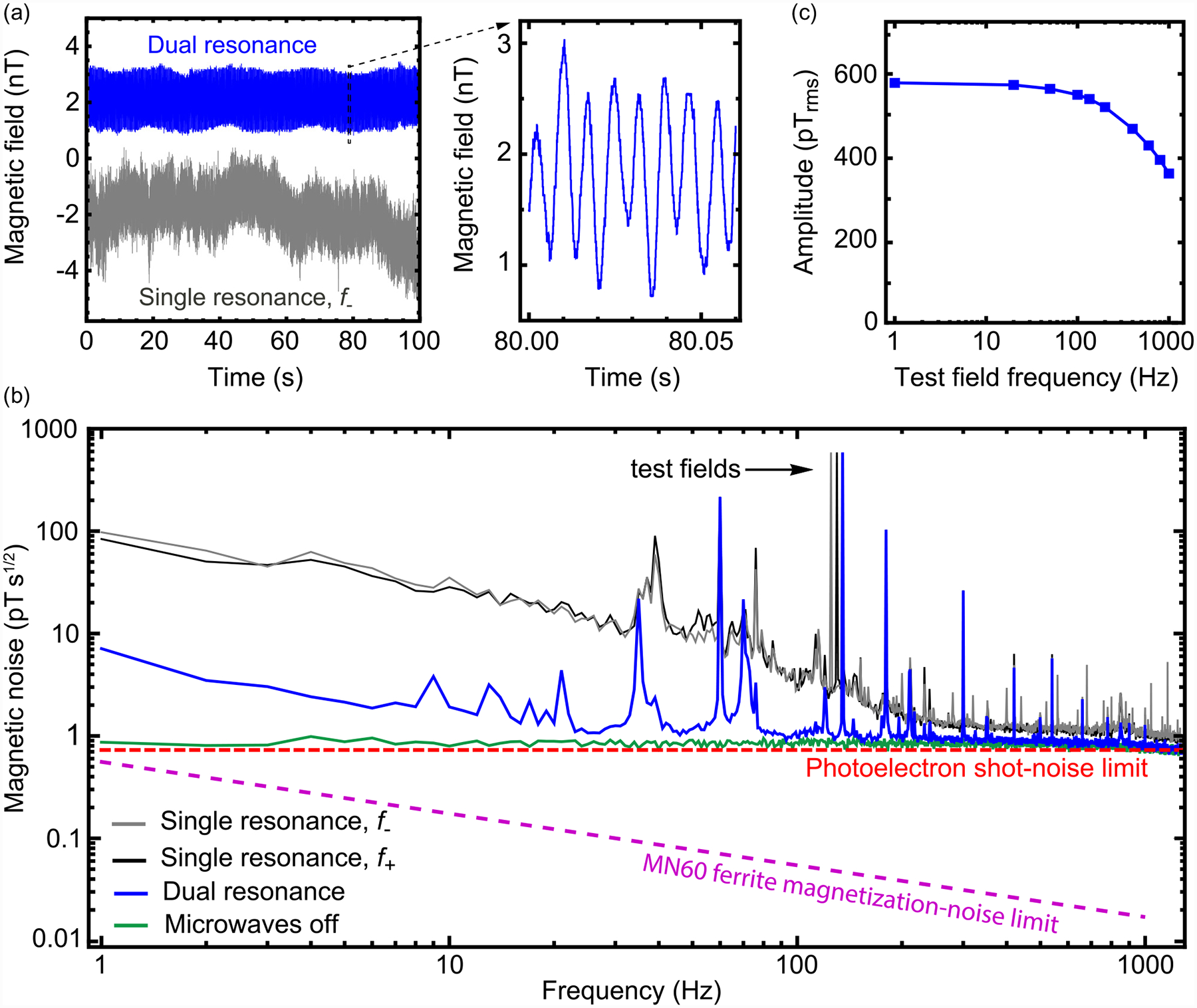
Sub-picotesla diamond magnetometry: (a) Time-domain lock-in signals for single-resonance (*f*_−_) and dual-resonance modulation. Throughout, *f*_mod_ = 15 kHz and the lock-in uses a 12 dB/octave low-pass filter with a 100 μs time constant. The adjacent plot is a zoom of the dual-resonance signal where the 580 pTrms test field at 135 Hz can be seen. The test-field frequency for *f*_+_ and *f*_−_ single-resonance experiments were 125 and 130 Hz, respectively, with the same 580 pTrms amplitude. (b) Magnetic noise spectra of single-resonance (two shades of gray) and dual-resonance (blue) signals. A reference spectrum obtained with microwaves turned off (green) shows noise from the unmodulated photodetector signal. Each spectrum was obtained by dividing a 100-s data set into one hundred 1-s segments, taking the absolute value of the Fourier transform of each segment, and then averaging the Fourier transforms together. Spectra were normalized such that the test field amplitudes matched the calibrated 580 pTrms values (Appendix H). The dashed red line is the projected value of *η*_psn_ for dual-resonance magnetometry (Appendix M). The dashed magenta line is the calculated thermal magnetization noise produced by the ferrite cones (Appendix P). (c) Frequency dependence of the test field amplitude measured by dual-resonance magnetometry.

We next show that the combination of flux concentration and dual-resonance modulation enables diamond magnetometry with sub-pT s^1/2^ sensitivity over a broad frequency range. A 1.73 μT bias field and 580 pTrms oscillating test field in the 125–135 Hz range were applied via the Helmholtz coils. The lock-in signal was continuously recorded for 100 s using either dual-resonance or single-resonance modulation. Figure 4(a) shows the magnetometer signals as a function of time. For single-resonance modulation, the signals undergo low-frequency drifts, likely due to thermal shifts of *D*(Δ*T*). These drifts are largely absent for dual-resonance modulation.

Figure 4(b) shows the magnetic noise spectrum for the different modulation techniques. In addition to the calibrated test field signals, numerous peaks appear for both single and dual-resonance modulation. We attribute these peaks to ambient magnetic noise that is not sufficiently attenuated by the single-layer mu-metal shield. In regions without peaks, the noise floor for single-resonance magnetometry is ~1.5 pT s^1/2^ for frequencies ≿300 Hz, but it exhibits nearly 1/*f* behavior for lower frequencies. On the other hand, the noise floor for dual-resonance magnetometry is ~0.9 pT s^1/2^ for frequenciesv ≿100 Hz and remains at this level, to within a factor of two, for frequencies down to ~10 Hz. The remaining noise below 10 Hz may be due to imperfect cancellation of the *D*(Δ*T*) thermal shifts or from temporal variation of *ϵ* due to either thermal variation in the gap length, *δ* (Appendix R), or ferrite permeability (Appendix S). For reference, a spectrum obtained with the microwaves turned off is also shown. It features a constant noise floor of ~0.8 pT s^1/2^ throughout the 1–1000 Hz frequency range. This level is consistent with the projected photoelectron shot-noise limit, *η*_psn_ = 0.72 pT s^1/2^, which was calculated based on the average photocurrent and lock-in slope (Appendix M).

The frequency response of the magnetometer was determined by recording magnetic spectra at different test-field frequencies, while holding the amplitude of the driving current constant. Figure 4(c) plots the test-field amplitude, recorded by dual-resonance diamond magnetometry, as a function of frequency. The amplitude decays by less than a factor of two over the 1–1000 Hz range. The observed decay is due to a combination of the lock-in amplifier’s low-pass filter and a frequency-dependent magnetic field attenuation due to metal components within the Helmholtz coils (Appendix H).

**FIG. 5.**
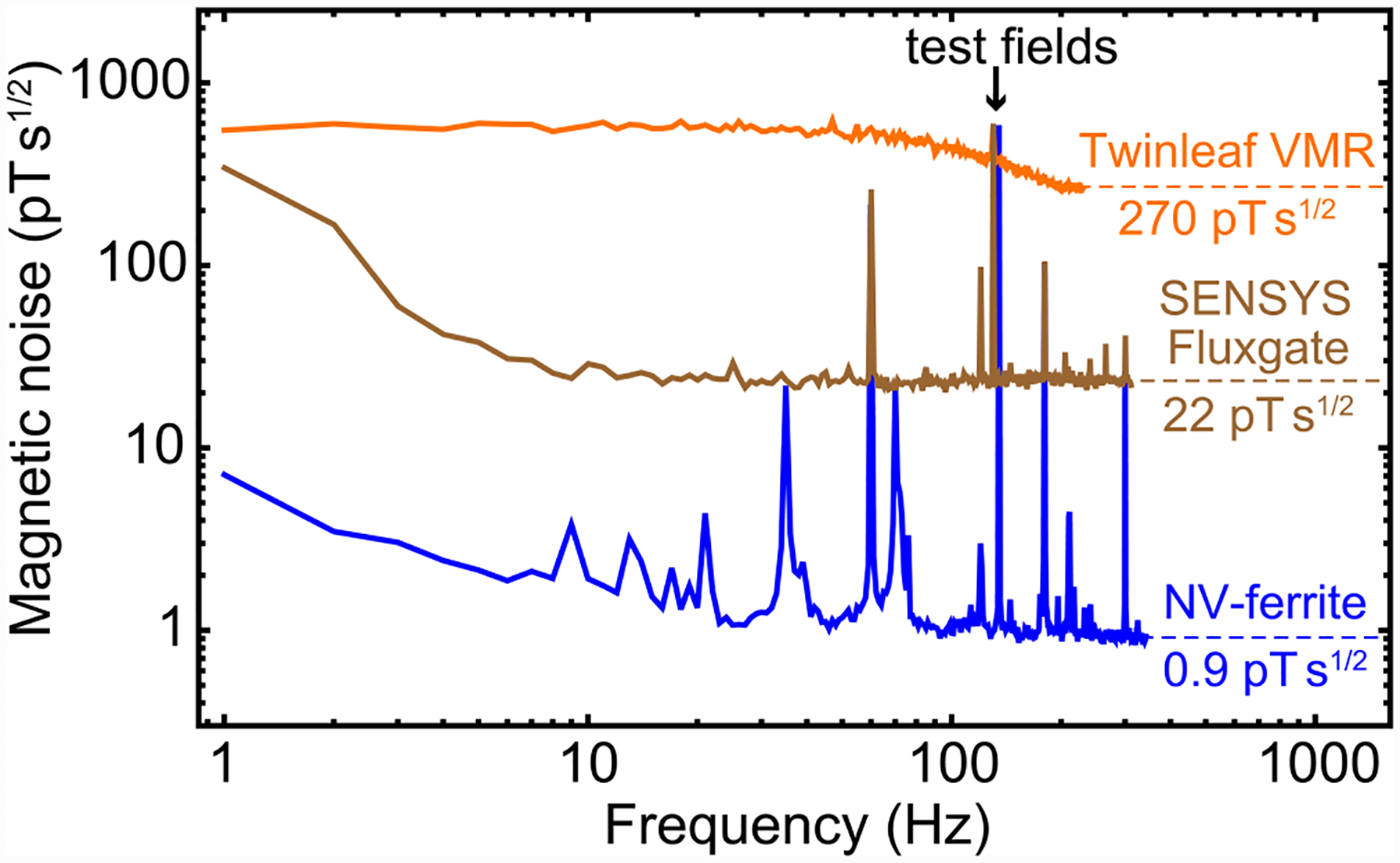
Magnetometer comparison: Magnetic noise spectra of a commercial magnetoresistive magnetometer (Twinleaf VMR), fluxgate magnetometer (SENSYS FGM-100) and our dual-resonance NV-ferrite magnetometer reproduced from Fig. 4(b). Each magnetometer was placed in a similar location within the experimental apparatus and subject to the same bias and test field amplitudes. The test field frequency was 130 Hz for both commercial sensors and 135 Hz for NV-ferrite. The manufacturer-specified sensitivities are $300\text{ pT}/\sqrt{\text{Hz}}$ and $10\text{ pT}/\sqrt{\text{Hz}}$ for the VMR and fluxgate, respectively.

Finally, we compared the performance of our magnetometer with two commercial vector sensors: a magnetoresistive magnetometer and a fluxgate magnetometer. Figure 5 shows the magnetic noise spectra obtained under comparable experimental conditions. Evidently, the NV-ferrite magnetometer outperforms these commercial magnetometers throughout the frequency range. The present sensitivity (≾1 pT s^1/2^) is comparable to that of commercial scalar alkali-vapor magnetometers that operate in μT bias fields. More sensitive (≾0.02 pT s^1/2^) vector alkali-vapor magnetometers are available, but they require bias fields ≾0.1 μT. SQUID magnetometers also offer superior sensitivity, but they require cryogenics.

## IV. DISCUSSION AND CONCLUSION

The demonstration of broadband, sub-picotesla diamond magnetometry is a significant step towards applications in precision navigation, geoscience, and medical imaging. Since only 200 mW of laser power and 20 mW of microwave power were used, the device holds promise for future miniaturization and parallelization efforts. Moreover, our magnetometer operates at microtesla ambient fields and its broad spin linewidth and small size make it relatively insensitive to field gradients. This raises the intriguing possibility of operating in Earth’s magnetic field (~50 μT) without additional bias fields or compensation coils.

Our implementation used a commercially available, type Ib HPHT diamond processed using standard electron-irradiation and annealing treatments [25]. This material exhibits relatively broad ODMR resonances (Γ ≈ 9 MHz), which leads to a photoelectron-shot-noise-limited sensitivity of *η*_psn_ = 0.72 pT s^1/2^ even after the ~250-fold flux-concentrator field enhancement. State-of-the-art synthetic diamonds have recently been fabricated that feature several orders of magnitude narrower resonances [24,48]. The excitation photon-to-photoelectron conversion efficiency in our experiments (*ξ* ≈ 10^−2^) could also be improved by at least an order of magnitude with optimized collection optics [17]. With these additions, *η*_psn_ could be further improved by several orders of magnitude [Eq. (1)]. Such an improvement in sensitivity is necessary for diamond magnetometers to compete with SQUID and alkali-vapor magnetometers in the most demanding applications such as MEG. However, at this level, thermal magnetization noise intrinsic to the flux concentrators becomes relevant.

Thermal magnetic noise originating from dissipative materials can be estimated using fluctuation-dissipation methods [39,49]. The noise has contributions due to thermal eddy currents and magnetic domain fluctuations. As discussed in Appendix P, we find that thermal eddy currents in the ferrite cones produce an effective white magnetic noise of ~7 × 10^−5^ pT s^1/2^. This negligibly low noise level is a consequence of our choice of low-conductivity ferrite. On the other hand, thermal magnetization noise results in a larger, frequency-dependent magnetic noise. At 1 Hz, this noise is 0.5 pT s^1/2^, and it scales with frequency as *f*^−1/2^, reaching ~0.02 pT s^1/2^ at 1 kHz. This noise, shown in Fig. 4(b), is not a limiting factor in our experiments, but it may have implications for future optimization efforts. If a material with a lower relative loss factor could be identified, it would result in lower thermal magnetization noise (Appendix Q).

In summary, we have demonstrated a diamond magnetometer with a sensitivity of ~0.9 pT s^1/2^ over the 10–1000 Hz frequency range. The magnetometer operates at ambient temperature and uses 0.2 W of laser power. These improved sensor properties are enabled by the use of ferrite flux concentrators to amplify magnetic fields within the diamond sensor. Our results may be immediately relevant to applications in precision navigation, geoscience, and medical imaging. More broadly, the use of micro-structured magnetic materials to manipulate magnetic fields offers a new degree of freedom for the design of diamond quantum sensors, with potential applications in magnetic microscopy [6–13] and tests of fundamental physics [50].

## Figures and Tables

**FIG. 6.**
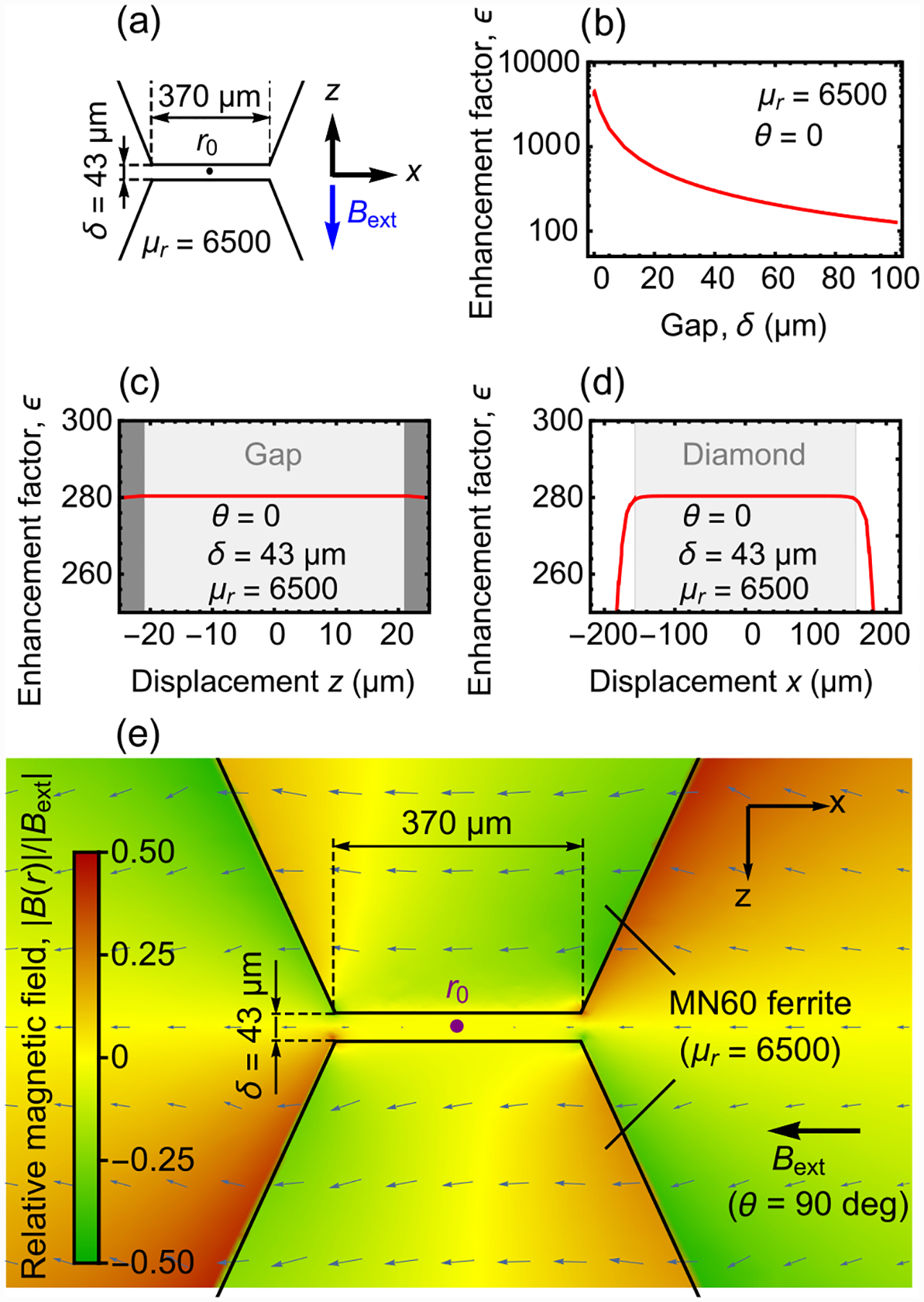
Enhancement factor and field homogeneity: (a) The model geometry. See Fig. 1(a) for additional dimensions. (b) Enhancement factor, *ϵ*, as a function of the gap length, *δ*. (c) Enhancement factor as a function of the axial displacement *z*. The gap is shaded in light gray, while the ferrite concentrators are shaded in dark gray. (d) Enhancement factor as a function of the transverse displacement *x*. (e) Simulated *x*-*z* plane cut of the relative magnetic field amplitude, |***B***(***r***)|/|***B***_**ext**_|, upon application of ***B***_**ext**_ at *θ* = 90°. See Fig. 1(b) for the case when ***B***_**ext**_ is applied at *θ* = 0.

**FIG. 7.**
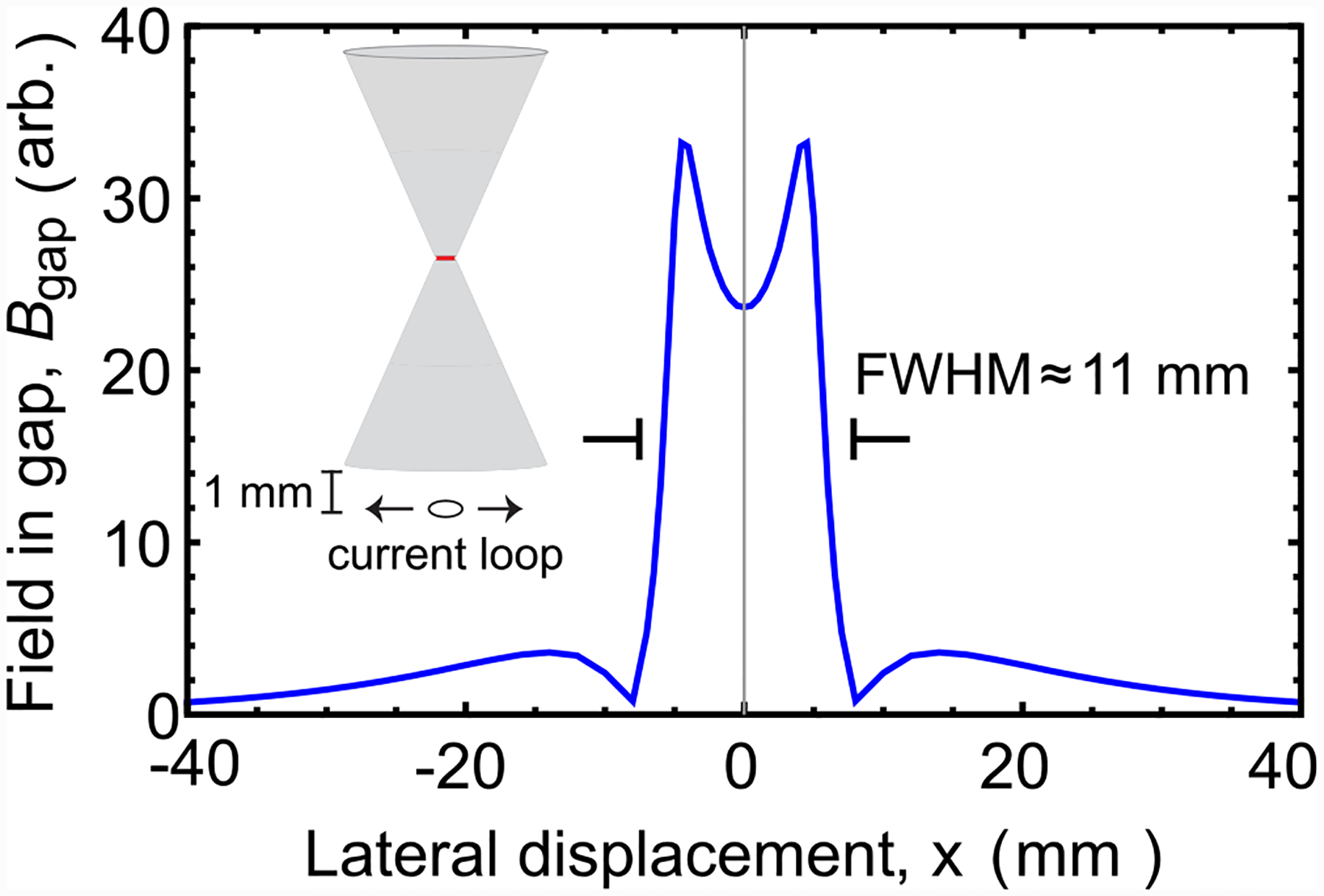
Flux concentrator point spread function: The value of *B*_gap_ due to a small current loop located below the device is recorded as a function of lateral displacement. The resulting field profile has a FWHM linewidth of ~11 mm. Inset: geometry for scanning.

**FIG. 8.**
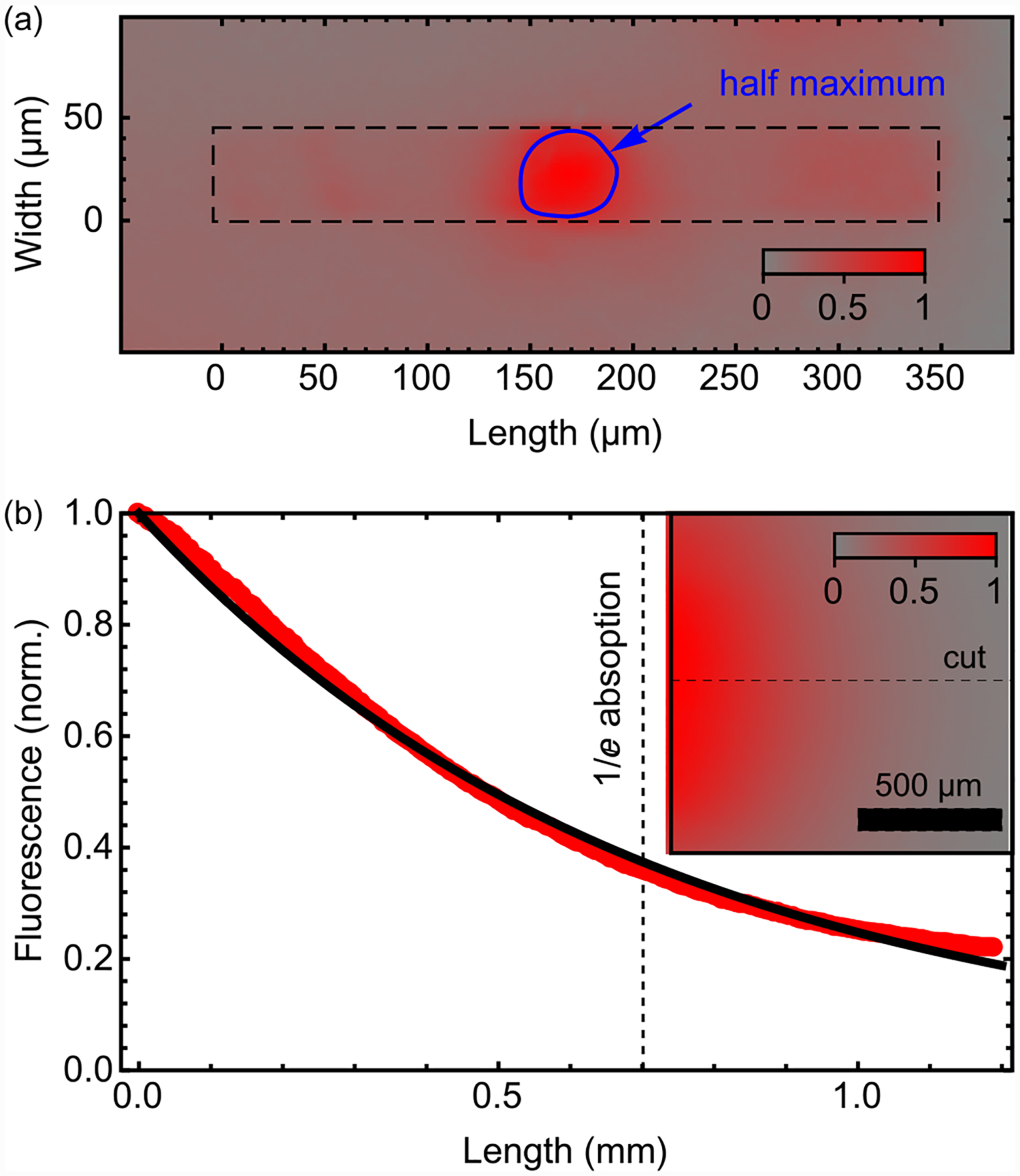
Beam profile and absorption length: (a) Image of the fluorescence spot at the entrance edge of the diamond membrane. The FWHM spot diameter is ~40 μm. The dashed lines indicate the approximate edges of the diamond. (b) Fluorescence intensity produced by a ~1 mm diameter laser beam entering the edge of a diamond membrane. The inset shows a fluorescence image of the top face. Red markers depict the normalized fluorescence intensity along the cut shown by the dashed line in the inset. The black solid line is an exponential fit, revealing a 1/*e* absorption length of 0.7 mm.

**FIG. 9.**
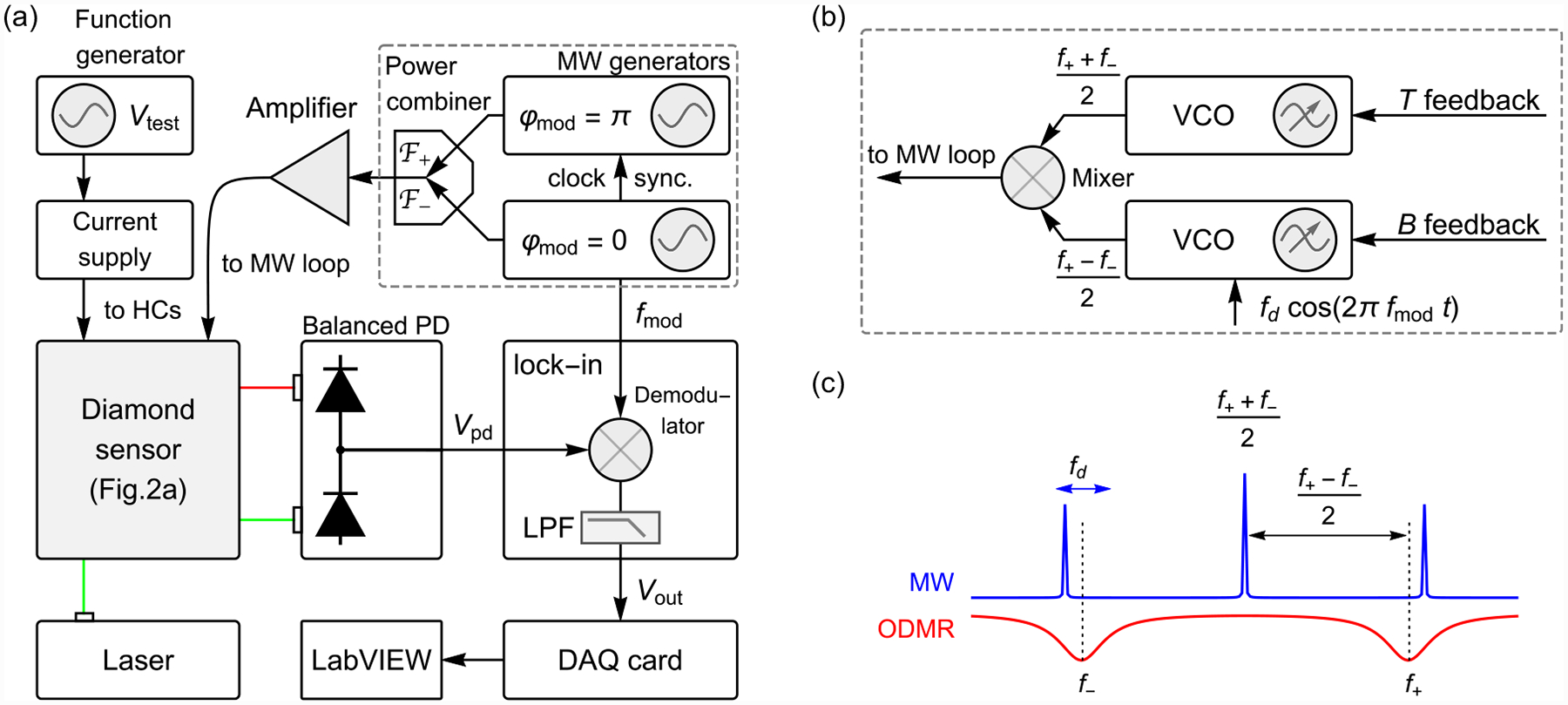
Electronics: (a) Schematic of the electronics portion of the experimental apparatus. *V*_pd_ is the photodetector signal, *V*_out_ is the lock-in amplifier’s in-phase output signal, *V*_test_ is the test signal waveform, and *f*_mod_ is the modulation frequency. (b) Alternative electronic scheme for dual-resonance microwave signal generation and feedback. A voltage-controlled oscillator (VCO) produces a carrier frequency *f*_car_ = (*f*_+_ + *f*_−_)/2 ≈ *D*(Δ*T*) that is mixed with the signal from a second VCO with frequency *f*_diff_ = (*f*_+_ − *f*_−_)/2, creating two sidebands at the ODMR frequencies. The sideband frequencies are modulated by adding a reference signal *f*_d_ cos (2*π f*_mod_*t*) to the second VCO. This arrangement allows for rapid feedback to correct for temperature and magnetic field drifts by adjusting the bias voltage to the VCOs. (c) Microwave signal spectrum resulting from the alternative electronics scheme in (b). A typical ODMR spectrum is shown in red for reference. DAQ: data acquisition card; HC: Helmholtz coils; LPF: low-pass filter; MW: microwave; PD: photodetector.

**FIG. 10.**
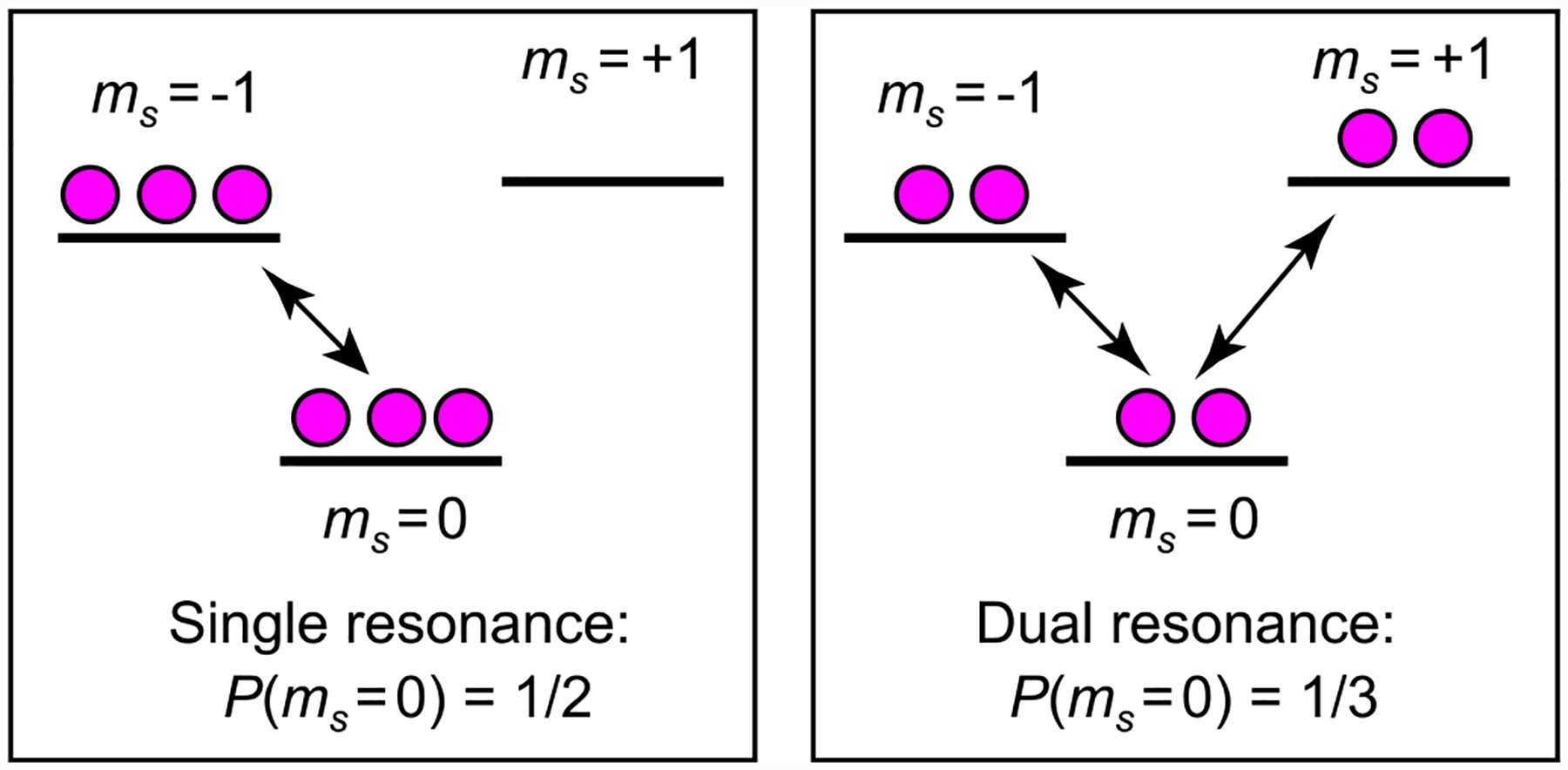
Single and dual-resonance spin populations: NV spin level populations, represented by the number of magenta circles, are shown under single-resonance and dual-resonance microwave excitation.

**FIG. 11.**
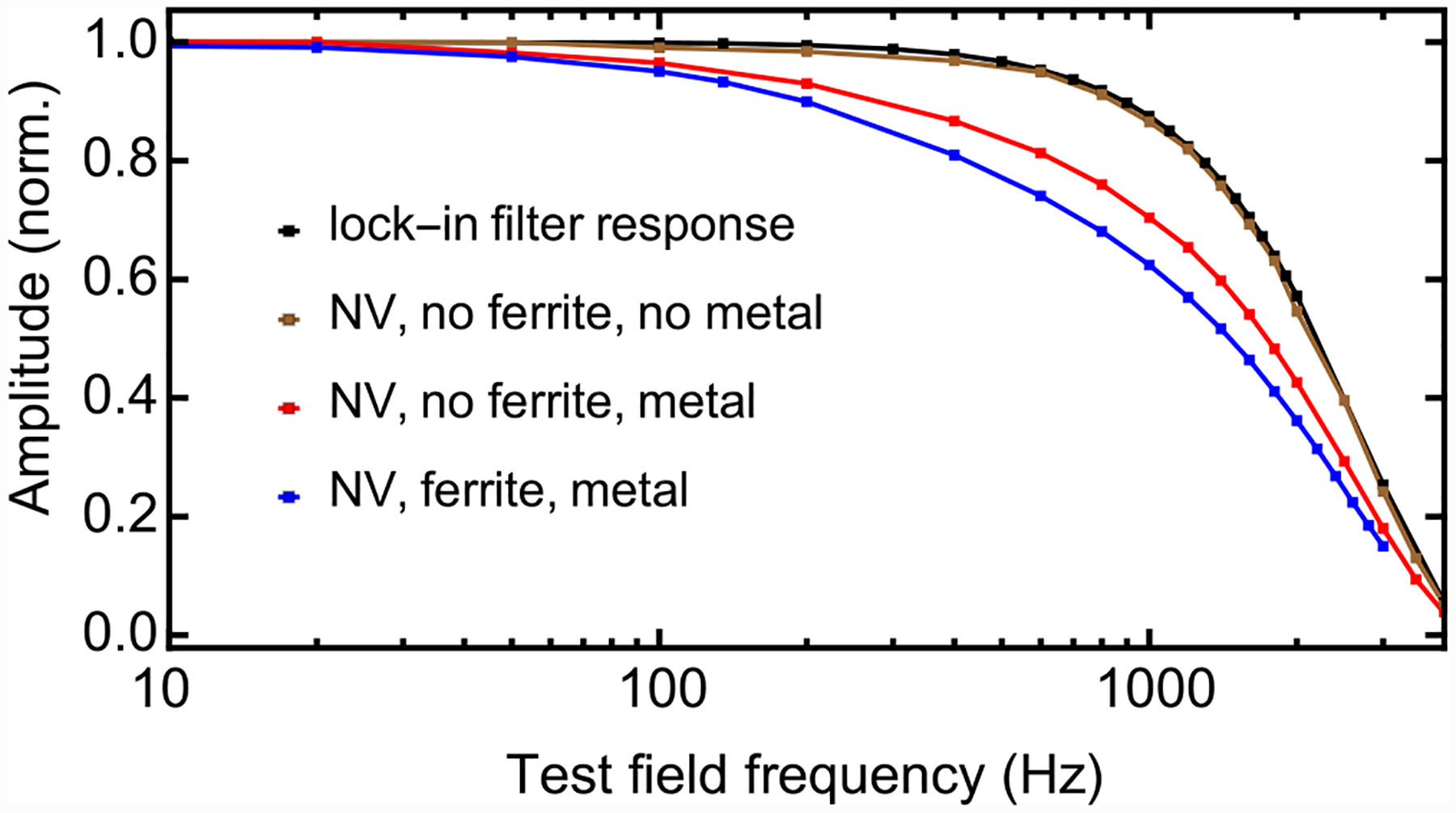
Frequency response of different magnetometer configurations: The blue trace is the normalized magnetometer frequency response, reproduced from Fig. 4(c). The red trace is the same NV magnetometer setup except without the ferrite cones. The brown trace is the NV magnetometer without ferrite cones and with metal components (Fig. 12) removed from the interior of the Helmholtz coils. The black trace is the lock-in filter response as measured by amplitude-modulated voltage inputs.

**FIG. 12.**
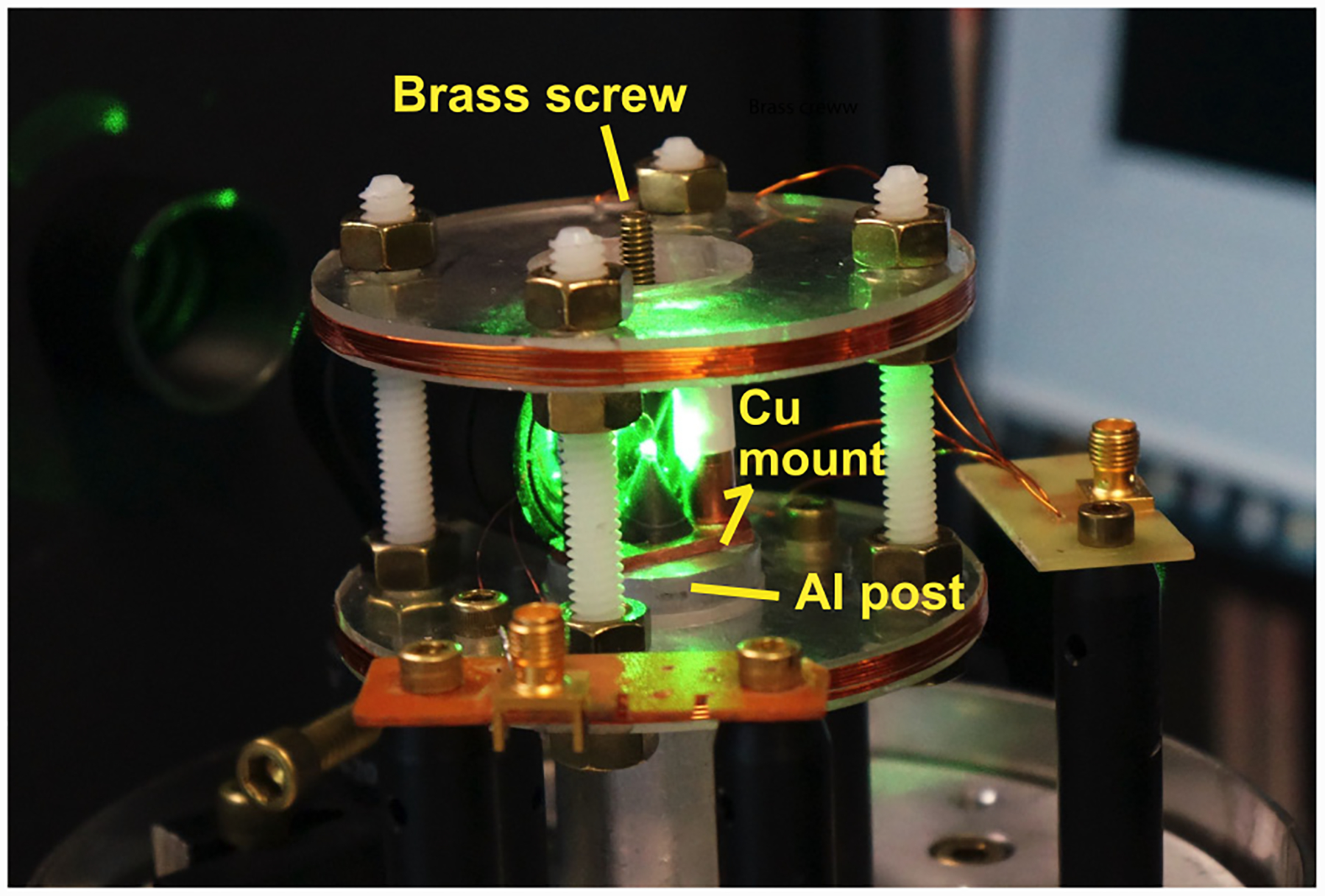
Photo of apparatus with shield removed: Metal mounting components that were removed to generate the data in Fig. 11 are labeled. The brass screw was used for mounting to a translation stage during initial alignment (Appendix C). Other unlabeled metal parts, such as brass nuts, were not found to contribute to the frequency-dependent magnetic field attenuation.

**FIG. 13.**
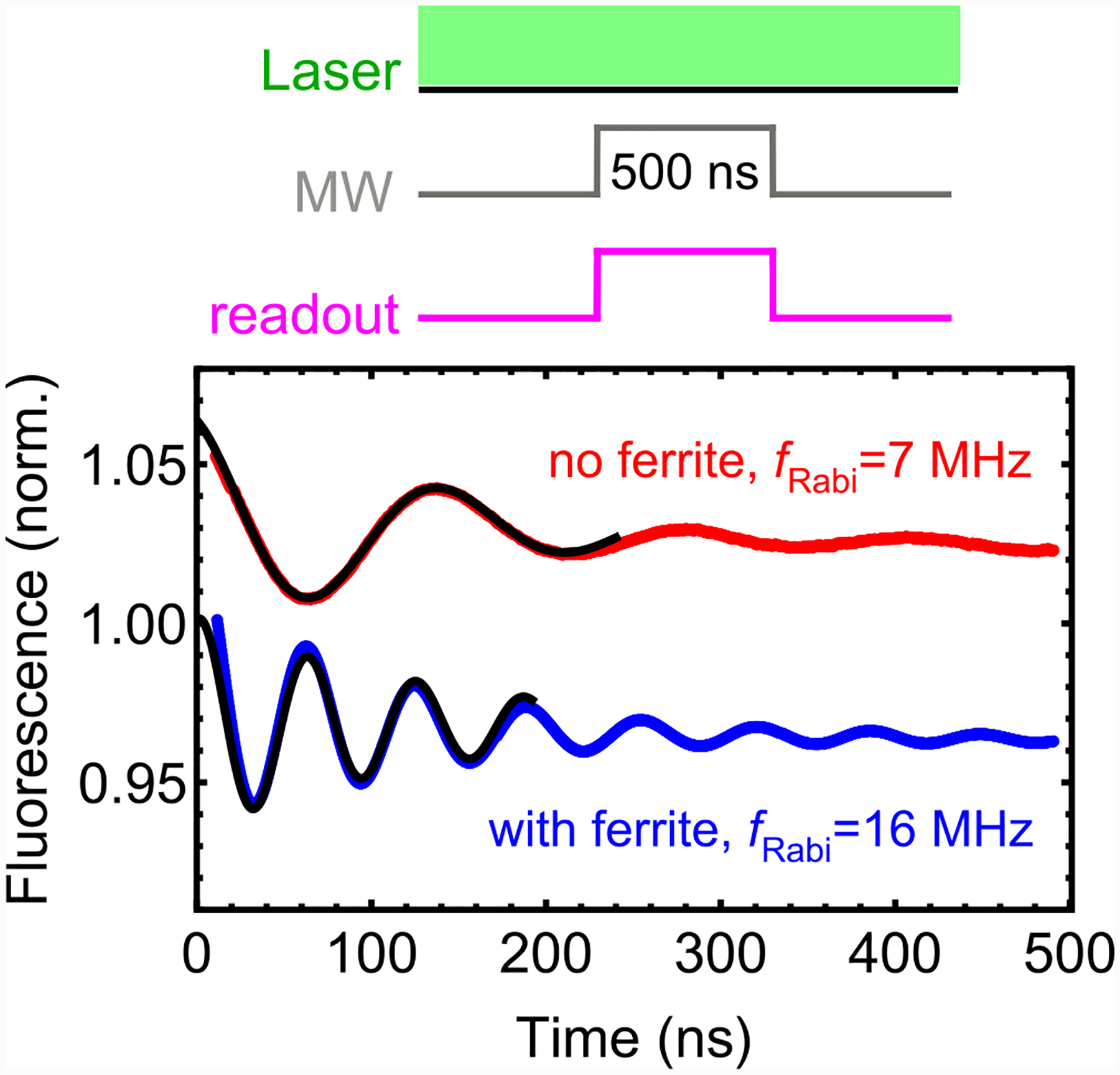
Rabi frequency with and without ferrite: (top) Protocol used to observe continuous-wave Rabi oscillations. (bottom) Rabi oscillations observed with and without ferrite cones (the setup was identical otherwise). Black solid curves are fits to an exponentially damped sinusoidal function revealing *f*_Rabi_ = 16 MHz with ferrite and *f*_Rabi_ = 7 MHz without ferrite. A microwave power of ~10 W was used for both traces in order to clearly visualize the Rabi oscillations.

**FIG. 14.**
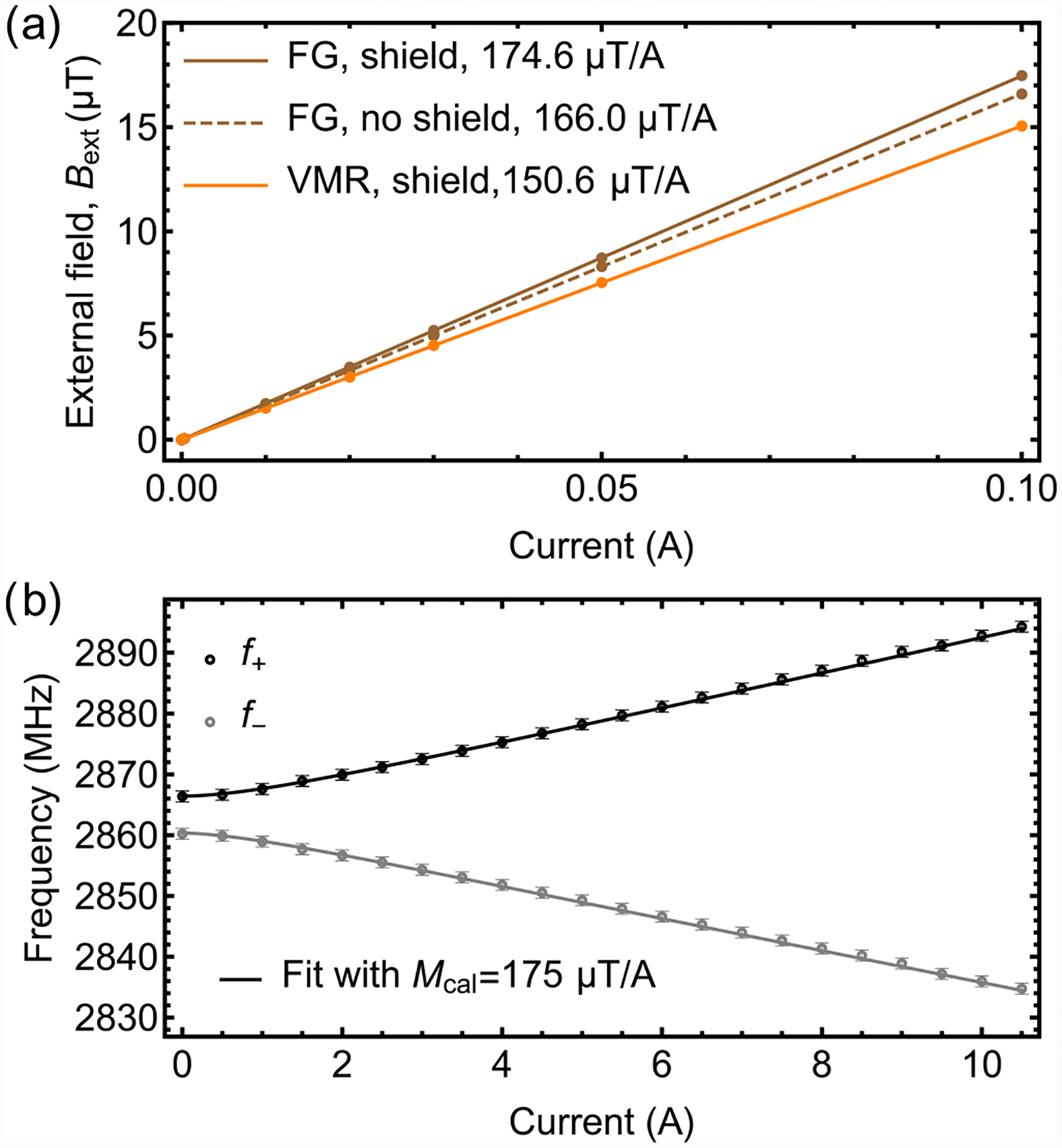
Helmholtz coils current calibration: (a) Helmholtz coils current calibration performed with two commercial vector magnetometers. FG: SENSYS FGM3D/100 fluxgate magnetometer; VMR: Twinleaf VMR magnetometer. (b) NV ODMR frequencies versus current in the Helmholtz coils. Solid lines are a fit using Eq. (A2), where *M*_cal_ = 175 μT/A is the fit parameter.

**FIG. 15.**
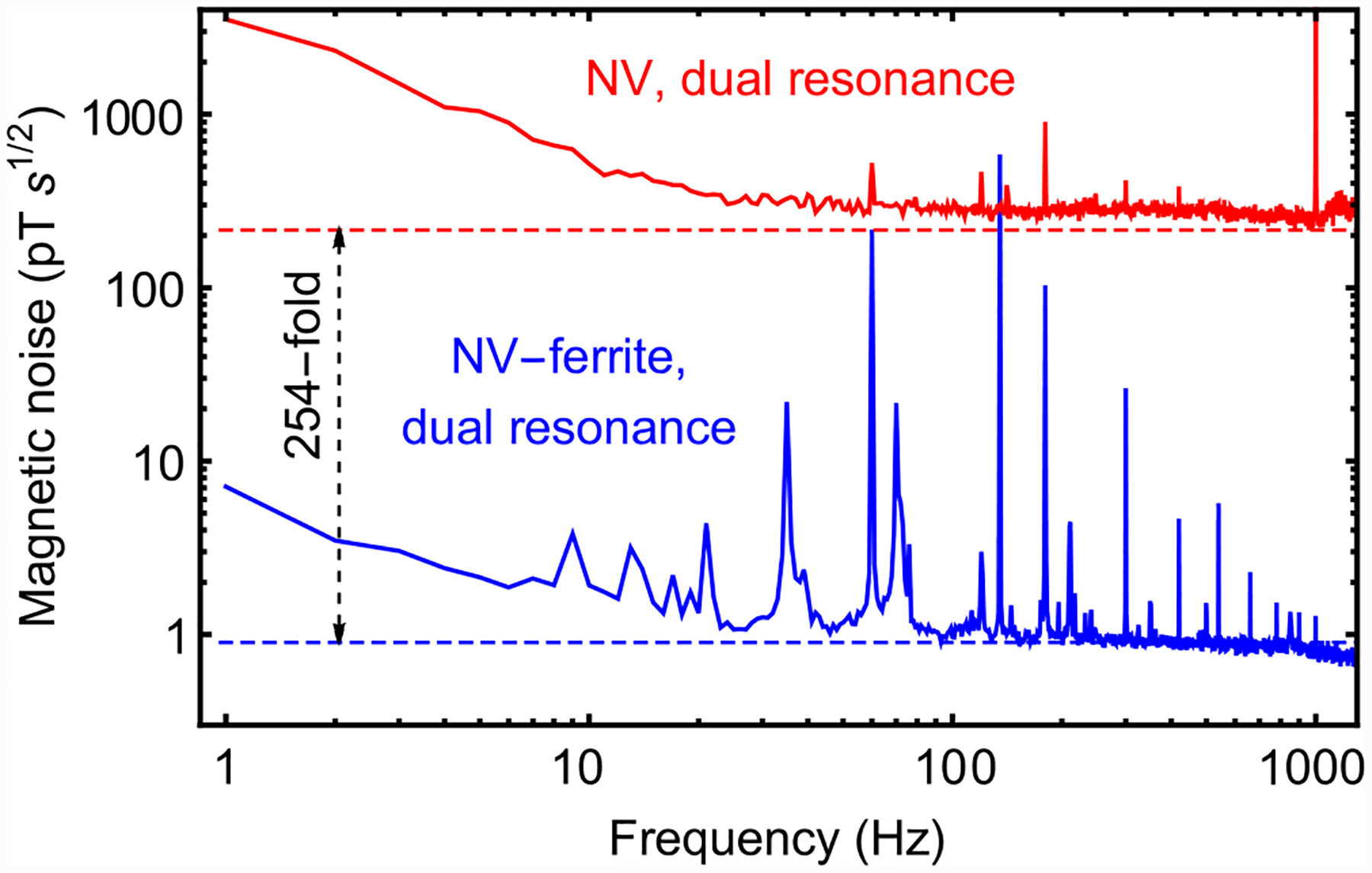
Sensitivity with and without ferrite cones: Magnetic noise spectra for dual-resonance magnetometry with (blue) and without (red) the ferrite cones.

**FIG. 16.**
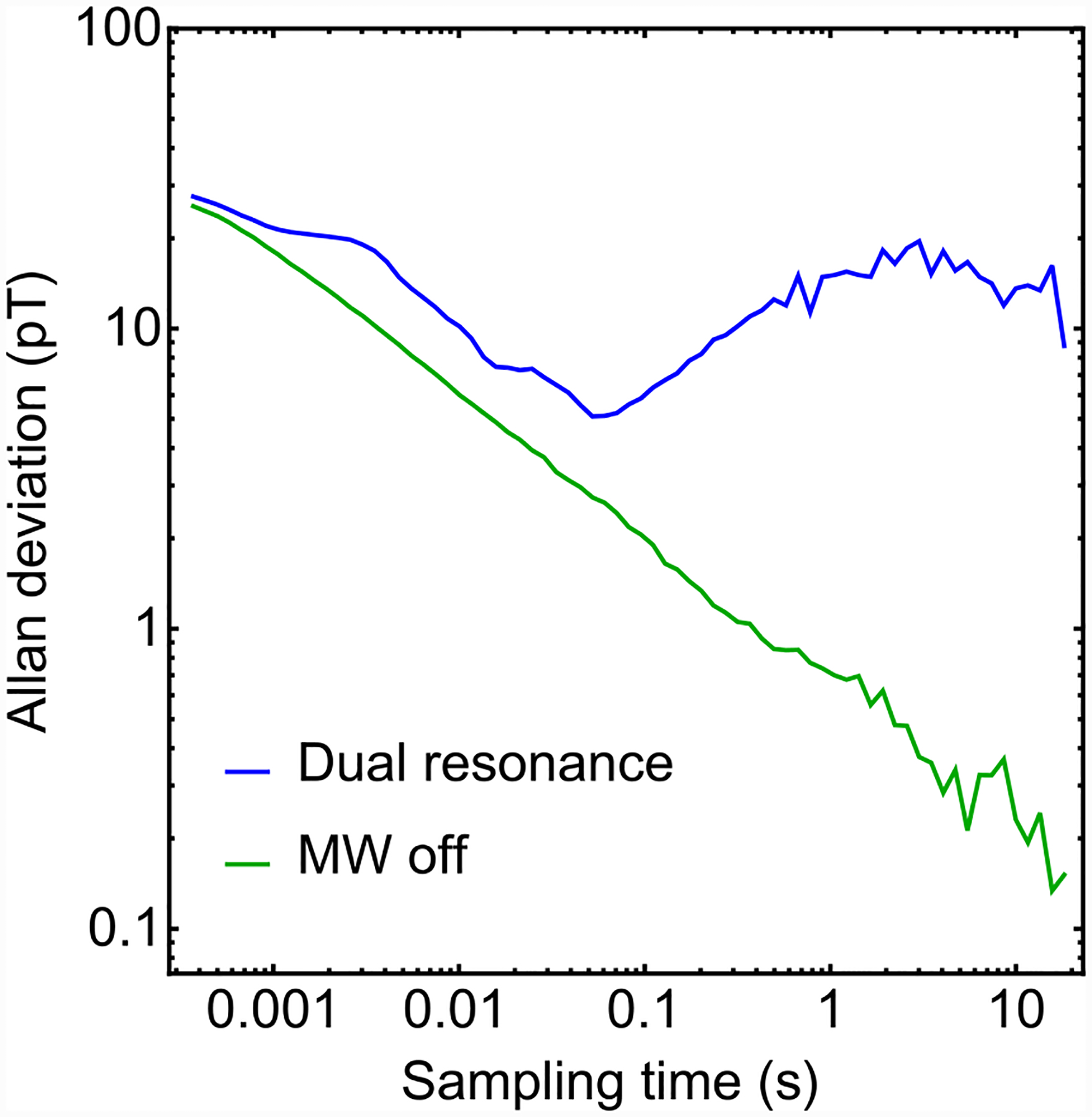
Allan deviations: Absolute Allan deviations of dual-resonance (blue), and microwaves-off (green) magnetometer signals. The original time-series data are the same as those studied in Fig. 4.

**FIG. 17.**
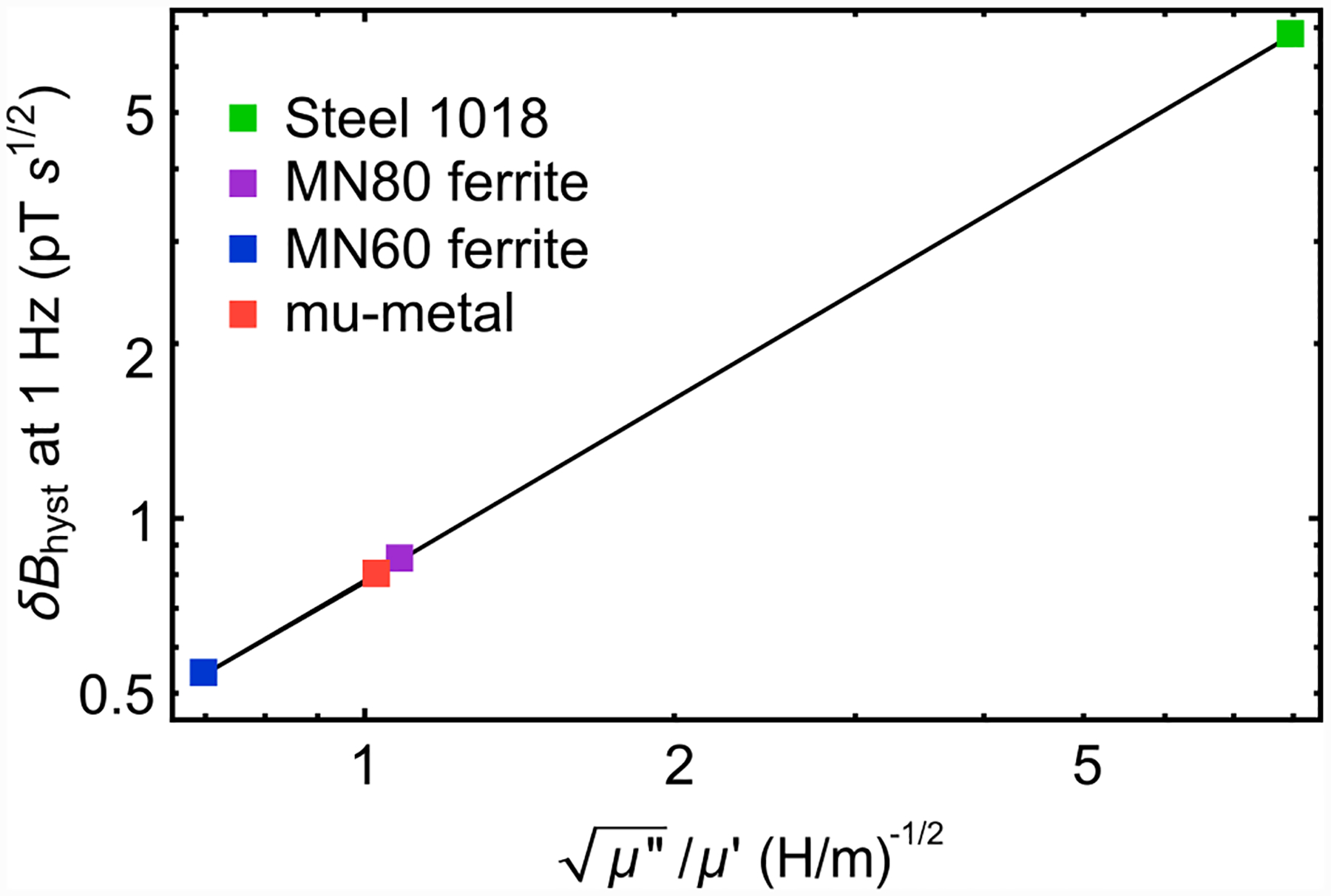
Magnetization noise vs relative loss factor: Calculated hysteresis magnetic noise (at 1 Hz) as a function of the square root of the relative loss factor, $\sqrt{\mu^{\prime \prime }}/\mu^{\prime }$ in four magnetic materials.

**FIG. 18.**
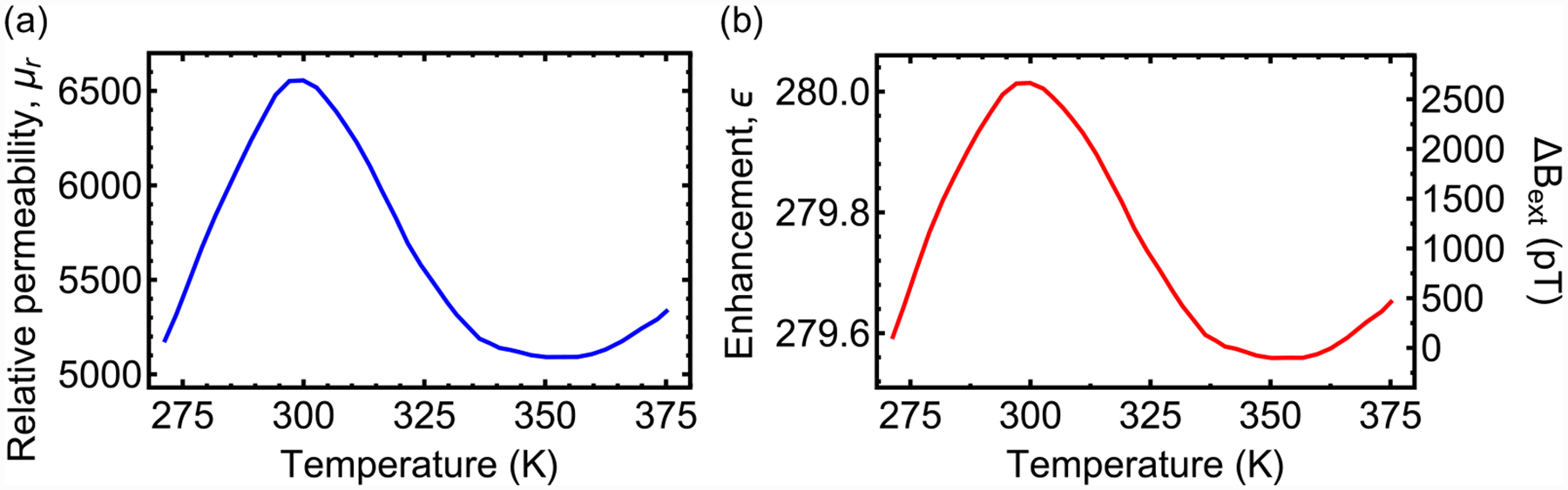
Thermal variation of ferrite permeability: (a) MN60 ferrite relative permeability as a function of temperature (as specified by Ceramic Magnetics, Inc.). (b) Simulated enhancement factor as a function of temperature, using the data in part (a). The right vertical axis shows the resulting change in magnetometer reading assuming *B*_ext_ = 1.7 μT.

**TABLE I.** Thermal magnetic noise for different cone materials: Magnetic noise arising from Hysteresis and Johnson noise were numerically calculated by the method described in Refs. [39,49] using finite-element methods. The values of *μ*′, *μ*″, and *σ* are taken from references: low-carbon steel 1018 [55], MnZn ferrite MN80 [56], MnZn ferrite MN60 [39], and mu-metal [39]. Note that *μ*′ and *μ*″ are in general frequency dependent. Here we take the values for the lowest reported frequency and assume that the response is relatively flat below 1 kHz. The enhancement *ϵ* is determined from magnetostatic simulations as in Fig. 1. The effective external magnetic noises *δB*_ext_ = *δB*_gap_/*ϵ* are defined by Eqs. (P1) and (P2). *δB*_ext_ is reported at 1 Hz. It scales with frequency as *f*^−1/2^.

Material	*μ*′/*μ*_0_	*μ*″/*μ*_0_	*σ* (S/m)	Enhancement, *ϵ*	δ*B*_hyst_ (1 Hz), pT s^1/2^	δ*B*_eddy_, pT s^1/2^
Steel 1018	250	5	5.18×10^6^	223	6.8	0.4
MnZn MN80	2030	6.1	0.2	251	0.85	0.00007
MnZn MN60	6500	26	0.2	254	0.54	0.00007
mu-metal	30 000	1200	1.6×10^6^	255	0.8	0.2

## APPENDIX A: NV ELECTRON SPIN HAMILTONIAN

Neglecting hyperfine coupling (which is not resolved in our experiments), the NV ground-state electron spin Hamiltonian can be written as [51]

(A1) $$\frac{\hat{H}}{h}=DS_{z^{\prime }}^{2}+E\left(S_{x^{\prime }}^{2}-S_{y^{\prime }}^{2}\right)+\gamma_{\text{nv}}B\cdot S,$$

where *h* is Planck’s constant, *γ*_nv_ = 28.03 GHz/T is the NV gyromagnetic ratio, and *E* ≈ 3 MHz is the transverse zero-field splitting parameter. The axial zero-field splitting parameter, *D* ≈ 2862 MHz, is temperature dependent, as discussed in Appendix D. *S* = (*S*_*x*′_*, S*_*y*′_*, S*_*z*′_) are dimensionless electron spin operators, and the *z*′ direction is parallel to the NV symmetry axis. For a magnetic field of amplitude *B*_gap_ applied normal to a diamond with [100] faces, the Hamiltonian for NV centers aligned along any of the four possible axes is the same. In matrix form, it is

(A2) $$\frac{\hat{H}}{h}=\left(\begin{array}{lll} D+\frac{\gamma_{\text{nv}}B_{\text{gap}}}{\sqrt{3}} & \frac{\gamma_{\text{nv}}B_{\text{gap}}}{\sqrt{3}} & E\\ \frac{\gamma_{\text{nv}}B_{\text{gap}}}{\sqrt{3}} & 0 & \frac{\gamma_{\text{nv}}B_{\text{gap}}}{\sqrt{3}}\\ E & \frac{\gamma_{\text{nv}}B_{\text{gap}}}{\sqrt{3}} & D-\frac{\gamma_{\text{nv}}B_{\text{gap}}}{\sqrt{3}} \end{array}\right),$$

The eigenstates and eigenfrequencies can be found by diagonalizing the Hamiltonian. The two microwave transition frequencies observed in our experiments, *f*_±_, correspond to the frequency differences between the eigenstate with largely *m*_*s*_ = 0 character and the eigenstates with largely *m*_*s*_ = ±1 character. We used this Hamiltonian to fit the *f*_±_ versus= *B*_ext_ data in Fig. 2(c). We assumed *B*_gap_ = *ϵB*_ext_ and used solutions to Eq. (A2) to fit for *ϵ* = 254. The values of *E* and *D* were determined separately from low-field ODMR data and were not fit parameters.

Note that Eq. (3), which approximates *f*_±_ as being linearly dependent on *B*_ext_, is merely a convenient approximation. As can be seen in Fig. 2(c), the exact values of *f*_±_ are generally nonlinear functions of *B*_ext_. This is especially pronounced near zero field, *ϵ*|*B*_ext_|≾ *E*/*γ*_nv_ ≈ 0.1 mT, where *f*_±_ undergo an avoided crossing, and also at high field, where mixing due to transverse fields produces nonlinear dependence. However, for magnetic fields 0.5 mT ≾*ϵB*_ext_ ≾ 5 mT, the transition frequencies *f*_±_ are approximately linear in *B*_ext_.

## APPENDIX B: FLUX CONCENTRATOR SIMULATIONS

Our flux concentrator model and simulations are described in Sec. II and Fig. 1. Here we describe supplementary results demonstrating the field homogeneity in the gap, the enhancement factor as the gap length approaches zero, and the approximate point spread function. Figure 6(a) describes the geometry used for the simulations. Figure 6(b) shows the enhancement factor as a function of *δ*, with the range extending to *δ* ≈ 0. The largest enhancement factors are observed for small gaps, approaching *ϵ* = 5000 for *δ* = 0. We chose a gap of *δ* ≈ 43 μm in our experiments as a compromise that offers moderate enhancement (*ϵ* ≈ 250) while still providing substantial optical access and straightforward fabrication and construction.

To visualize the homogeneity of the magnetic field within the gap, we plot line cuts of the relative field amplitude along the axial and transverse directions. Figure 6(c) shows the relative magnetic field along the cone symmetry axis. Figure 6(d) shows the relative field along a transverse line passing through ***r***_**0**_. Both plots predict a high degree of magnetic field homogeneity; residual variations of the relative field are ≾1% throughout the region filled by the diamond membrane.

For reference we also simulated the magnetic field distribution when a magnetic field s applied transverse to the cone axis. Figure 6(e) shows simulated *x*-*z* plane cut of the relative magnetic field amplitude upon application of the external magnetic field along the transverse directions. The field is uniformly suppressed throughout the gap.

Future NV-flux concentrator devices may involve the use of sensor arrays to perform imaging. While a detailed analysis of the design space for imaging applications is beyond the scope of this work, we performed simulations to estimate the point spread function of our device. A small (1-mm diameter) current loop was positioned to have an axial displacement of 1 mm below the base of the bottom cone. The magnetic field amplitude in the gap, *B*_gap_, was simulated as a function of the current loop’s lateral displacement, *x*. Figure 7 shows the resulting magnetic field profile. While it does not a have simple Gaussian shape, it can be approximated as having a full-width-at-half-maximum (FWHM) resolution of ~11 mm.

## APPENDIX C: EXPERIMENTAL SETUP: CONES

The ferrite cones were ordered from Precision Ferrites & Ceramics, Inc. The diamond membrane was glued on the tip of one of the ferrite cones with LOCTITE AA3494 UV-curing adhesive. The second cone with the microwave loop was mounted inside a metallic holder and micropositioned to contact the exposed face of the diamond membrane by use of a Thorlabs MicroBlock Compact Flexure Stage MBT616D. When in the desired position, the holder was glued to the support of the bottom cone by superglue, and then detached from the micropositioning stage.

## APPENDIX D: EXPERIMENTAL SETUP: OPTICS

To excite NV fluorescence, a Lighthouse Photonics Sprout-G laser is used to form a collimated beam of 532 nm light. The beam is focused with a 1-inch diameter, 0.79-NA aspheric condenser lens (Thorlabs ACL25416U-B) onto the edge of the diamond membrane. Fluorescence is collected by the same lens and is spectrally filtered by a Semrock FF560-FDi01–25 × 36 dichroic mirror. A second lens (1-inch diameter, 50-mm focal length) focuses the fluorescence onto a photodetector. The emission path length between aspheric condenser and final lens is ~175 mm. For magnetometry experiments, including all data in the figures in the main text, we used a Thorlabs PDB210A balanced photodetector with an active diameter of 5 mm. For beam characterization (Fig. 8), we used a CMOS image sensor, and for observing Rabi oscillations (Fig. 13 below), we used a Thorlabs PDA8A high-speed photodetector. Figure 8(a) shows an image of the fluorescence spot from the entrance edge of the diamond membrane. The FWHM spot diameter of ~40 μm was selected to match the diamond membrane thickness. It was adjusted by tailoring additional telescoping lenses in the excitation path.

With this optical system, we obtained a excitation photon-to-photoelectron conversion efficiency of *ξ* ≈ 0.01. The primary factors limiting *ξ* are due to the limited optical access afforded by the ferrite cones, loss of fluorescence exiting orthogonal faces of the diamond membrane, and incomplete absorption of the excitation beam within the diamond. To characterize the latter, we used a separate apparatus to image the fluorescence from the top face of a larger membrane [Fig. 8(b)]. This larger membrane was the starting piece from which we cut the smaller membrane used in magnetometry experiments. We found that the 1/*e* absorption length of this material is 0.7 mm. Thus we expect that only ~35% of the laser light was absorbed in the ~300 μm-long diamond membrane used in magnetometry experiments. This approximation neglects the effects of laser light that is reflected at the air-diamond interfaces.

The large absorbed optical power results in significant heating of the diamond membrane. The experimentally measured axial zero-field splitting parameter *D* ≈ 2862 MHz [Fig. 2(c)] indicates a local diamond temperature of ~385 K [42]. While the elevated temperature leads to a large shift in *D*, it does not significantly diminish the contrast or broaden the ODMR resonances. Future devices may employ active cooling or optimized heat sinks to reduce the diamond temperature. Fortunately, the microwave irradiation contributes only a negligible amount to the overall heating. By varying the microwave power, we found that the shift in *D* due to 20 mW of delivered microwave power was <0.1 MHz. This corresponds to a change in temperature of *<*1 °C.

Our optical collection setup results in an effective NV fluorescence collection volume that is the product of the excitation beam area, ~*π* × (20 μm)^2^, and the optical path length in the diamond, 300 μm. The justification for our assumption that we collect fluorescence from the entire length of the diamond is a combination of two factors. The first factor is that, while a reasonably high numerical aperture (NA = 0.79) aspheric lens is used for collection, the overall imaging system has a relatively low magnification (*M* = 3.1), and a large-area photodiode (5 mm diameter) is used for detection. This means that we still collect light from emitters with a large axial displacement from the focus. To verify this, we performed ray tracing using an ABCD matrix calculation of the marginal rays collected from point sources along the optical axis. We found that the axial collection range (defined as the range where the collection efficiency is at least half as much as at the focus) for this imaging system is >0.8 mm for point emitters in air. The second factor is that we expect the waveguiding effect of the diamond slab to make the emission appear even more localized [52]. Due to total internal reflections, the emission exits the diamond primarily out of the four edge facets. Each edge facet has a cross section of 40 μm × 300 μm, and we collect from only one edge. After 3.1× magnification, the image of the edge facet is still much ×smaller than the photodetector active area.

## APPENDIX E: EXPERIMENTAL SETUP: ELECTRONICS

Figure 9(a) shows a schematic of the electronic devices used in our experimental setup. Microwaves are supplied by two Stanford Research SG384 signal generators. The clocks of the generators are synchronized by passing the 10 MHz frequency reference output of one generator to the frequency reference input of the other. Both generators are configured to modulate the microwave frequency with a modulation frequency *f*_mod_ = 15 kHz and depth *f*_*d*_ = 3.3 MHz. In dual-resonance modulation, the signal generators are configured such that their modulation functions, $F_{\pm }$, have a relative *π* phase shift (see Sec. II). The signals from both generators are combined with a Mini-Circuits ZAPD-30-S+ 2-way power combiner, amplified by a Mini-Circuits amplifier ZHL-16W-43-S+, and finally delivered to a two-turn microwave loop made from polyurethane-enameled copper wire (38 AWG). Prior to performing dual-resonance magnetometry, the microwave powers for each *f*_±_ resonance were independently adjusted to give approximately the same lock-in slope [Fig. 3(c)].

The photodetector output signal, *V*_pd_, is fed to a Signal Recovery 7280 lock-in amplifier using 50 Ω termination. The lock-in multiplies *V*_pd_ by a reference signal, proportional to cos (2*π f*_mod_*t*), output from one of the signal generators. The demodulated signal is processed by the lock-in’s low-pass filter, which was set to 12 dB/octave with a 100 μs time constant. The lock-in amplifier’s in-phase component, *V*_out_, is digitized at 50 kS/s by a National Instrument USB-6361 data acquisition unit.

External fields, *B*_ext_, are produced by a pair of Helmholtz coils (radius: 38 mm) driven by a Twinleaf CSUA-50 current source. To create oscillating test signals, a Teledyne LeCroy WaveStation 2012 function generator provides a sinusoidal waveform, *V*_test_, to the modulation input of the current source. The same function generator was used to slowly sweep the magnetic field for the lock-in signals shown in Fig. 3(c) (in this case, no oscillating test signals were applied).

While our tabletop prototype uses scientific-grade microwave generators, a simpler system could be used to deliver the requisite dual-resonance microwave waveforms. Figure 9(b) shows an alternative scheme which uses only voltage-controlled oscillators and a mixer. This scheme has the benefit of allowing for rapid feedback to compensate for thermal and magnetic field drifts, which would enable a higher dynamic range [47].

## APPENDIX F: DUAL-RESONANCE MAGNETOMETRY

We perform our magnetometry experiments with a lock-in amplifier in order to reduce technical noise, particularly at low frequencies. Such noise could arise from a variety of sources, but a common source in NV magnetometry experiments is due to intensity fluctuations of the laser that are not fully canceled by balanced photodetection. The lock-in method allows us to tune our photodetector signal to a narrow frequency band, where such technical noise is minimal. In our experiments, this is accomplished by modulating the microwave frequency at a modulation frequency *f*_mod_ = 15 kHz and depth *f*_*d*_ = 3.3 MHz. The resulting photodetector signal, *V*_pd_, has components at *f*_mod_ and higher harmonics, in addition to the DC level. The lock-in amplifier isolates the component at *f*_mod_, in a phase-sensitive manner, by multiplying *V*_pd_ by a reference signal proportional to cos (2*π f*_mod_*t*). The product signal is passed through a low-pass filter, and the in-phase component, *V*_out_, serves as the magnetometer signal.

The lock-in signal, *V*_out_, can be converted to magnetic field units by one of two methods. In the first case, one can sweep the magnetic field and measure the dependence of *V*_out_ on *B*_ext_, as in Fig. 3(c). The slope can be used to infer the conversion of *V*_out_ to magnetic field units. This method works well provided that the slope never changes. In practice, the slope can change due to drifts of the laser or microwave powers. It also can’t account for any dependence of *V*_out_ on magnetic field frequency, as the slope is measured at DC. Thus, we always apply a calibrated oscillating test field and re-normalize our magnetometer conversion based on the observed amplitude. Typically the difference in conversion factors using the two methods is small (≾10%).

We now turn to describing the principle of dual-resonance magnetometry. In single-resonance magnetometry, the microwave frequency is modulated about one of the ODMR resonances (for example, *f*_+_) and demodulated at the same frequency. The in-phase lock-in output *V*_out_ is proportional to small deviations in *f*_+_. This allows one to infer both the magnitude and sign of changes in *f*_+_. If the relative phase between the microwave modulation function, $F_{+}$, and the reference signal were shifted by *π* radians, the magnitude of *V*_out_ would be the same but the sign would reverse.

In dual-resonance magnetometry, we exploit this feature of phase-sensitive detection. The microwave modulation function for one resonance has a *π* phase shift with respect to the modulation function of the second resonance. The reference signal has the phase of the first modulation function. In this way, if both *f*_+_ and *f*_−_ shift by equal amounts in the same direction [due to a change in *D*(Δ*T*)], their contributions to the lock-in signal cancel and *V*_out_ = 0. If *f*_+_ and *f*_−_ shift by equal amounts but in opposite directions (due to a −change in *B*_ext_), their contributions to the lock-in signal add together and *V*_out_ changes in proportion to their shift. In other words, the lock-in output is unaffected by thermal shifts of the NV spin levels (which shift *f*_+_ and *f*_−_ by equal amounts in the same direction), but it remains proportional to changes in magnetic field (which shift *f*_+_ and *f*_−_ by approximately equal amounts in opposite directions).

Note that dual-resonance modulation could also be used to make an NV thermometer which is unaffected by changes in magnetic field. This would be accomplished by applying the same modulation phase to both $F_{\pm }$ signals and monitoring the in-phase lock-in signal.

## APPENDIX G: SENSITIVITY ENHANCEMENT IN DUAL-RESONANCE MAGNETOMETRY

The dual-resonance magnetometry approach was primarily used because it is unaffected by thermal shifts of the NV spin levels. This enabled better low-frequency performance. However the dual-resonance approach also has a fundamental advantage in sensitivity for all frequencies. Compared to the single-resonance approach, it offers a ~4/3-fold improvement in photoelectron-shot-noise-limited sensitivity. This improvement comes about due to a ~4/3-fold increase in the ODMR contrast.

To understand where the factor of 4/3 arises, consider the limiting case when the microwave excitation rate is much larger than the optical excitation rate. In this regime, a resonant microwave field drives the spin levels it interacts with into a fully mixed state (Fig. 10). For single-resonance excitation, when the microwave field is on resonance, the probability that NV centers will be in the *m*_*s*_ = 0 level is *P*_0_ = 1/2. For dual-resonance excitation, both microwave transitions share the *m*_*s*_ = 0 level and thus *P*_0_ = 1/3 when both microwave fields are on resonance. Defining the fluorescence intensity of an NV center in the *m*_*s*_ = 0 level as *I*_0_ and the fluorescence intensity of an NV center in either of the *m*_*s*_ = ±1 levels as *I*_1_, the ODMR contrast is given by

(G1) $$C=\frac{I_{0}-\left[P_{0}I_{0}+\left(1-P_{0}\right)I_{1}\right]}{I_{0}}.$$

For the single-resonance case, the contrast is $C_{s}=\frac{1}{2}\frac{I_{0}-I_{1}}{I_{0}}$. In the dual-resonance case, the contrast is $C_{d}=\frac{2}{3}\frac{I_{0}-I_{1}}{I_{0}}$. The ratio is therefore *Cd*/*Cs* = 4/3. Since the photoelectron-shot-noise-limited sensitivity is proportional to 1/*C* [Eq. (1)], this corresponds to a 4*/*3 reduction in the magnetic noise floor.

To derive the factor of 4/3 we assumed that the microwave excitation rate was larger than the optical excitation rate. In experiments, we use 20 mW of microwave power. This corresponds to a microwave Rabi frequency of ~ 0.7 MHz (Appendix I) or a spin flip rate of ~1.4 × 10^6^ s^−1^. The optical intensity used in our experiments was *I*_opt_ ≈ 0.2 W/(40 μm)^2^ = 12.5 kW/cm^2^ (Appendix D). The NV absorption cross section at 532 nm is *σ*_nv_ ≈ 3 × 10^−17^ cm^2^ [25], so this corresponds to an optical excitation rate of *I*_*opt*_*σ*_nv_/*E*_ph_ ≈ 10 s^−1^. Thus, in our experiments, the microwave excitation rate is comparable to, or slightly larger than, the optical excitation rate. The improvement in dual-resonance sensitivity was thus not exactly 4/3, but it was close (~1.3). Another assumption that we implicitly made is that the ODMR linewidth is the same under single-resonance and dual-resonance excitation. This assumption is reasonably accurate in our experiments; see Fig. 3(c).

## APPENDIX H: MAGNETOMETER FREQUENCY RESPONSE

Figure 4(c) shows the amplitude of test fields, recorded by dual-resonance diamond magnetometry, as a function of their frequency. A moderate decay (~40%) of the signal amplitude was observed over the 1–1000 Hz range. In order to determine the causes of this signal decay, we performed a series of frequency-response measurements under different conditions.

Figure 11 shows the results of these experiments. In all cases we use *f*_mod_ = 15 kHz and the lock-in uses a 12 dB/octave low-pass filter with a time constant *τ*_li_ = 100 μs. We first isolated the lock-in amplifier’s frequency response by applying a sinusoidal voltage, oscillating at *f*_mo_d = 15 kHz, with an amplitude modulation of constant depth and variable modulation frequency. The resulting lock-in response is well described by a second-order Bessel filter with a cutoff frequency of 1/(2*πτ*_li_). While this filter is largely responsible for the magnetometer decay at frequencies ≿1 kHz, it can only account for a small fraction of the decay observed over the 1–1000 Hz range.

Next, we removed the ferrite cones from the assembly and performed dual-resonance magnetometry. The observed frequency response is similar to that observed with the ferrite cones in place. The decay is slightly less pronounced, but evidently the ferrite cones do not account for the observed decay.

Finally, we removed the metal mounting hardware used in the apparatus that were located within the Helmholtz coils (Fig. 12). We again performed dual-resonance diamond magnetometry without the ferrite cones in place. In this case, we observe a frequency response which is nearly identical to the lock-in amplifier’s frequency response.

We therefore conclude that metal components within the Helmholtz coils are responsible for most of the decay in the 1–1000 Hz range observed in Fig. 4(c). The lock-in amplifier’s low-pass filter contributes as well, but to a lesser degree. The ferrite cones may also contribute a small amount to the observed decay, but future work would be needed to isolate their response independently.

The frequency dependence of our magnetometer leaves an ambiguity as to how best to normalize the magnetic noise spectra in Fig. 4(b). As seen in Fig. 4(c), when we apply a test current which is expected to produce an amplitude of 580 pTrms, it produces the correct amplitude at 1 Hz, but at 125–135 Hz it produces an amplitude of ~540 pTrms. Since 125–135 Hz is the frequency range of the test fields applied in Fig. 4(b), we therefore had to decide whether to normalize the noise spectra so that the test-field peaks appeared at 580 pT s^1/2^ or ~540 pT s^1/2^. Conservatively, we chose the former. We multiplied each spectrum by 580/540 = 1.07, which raised the test field peaks to 580 pT s^1/2^ and also raised the noise floor by 7%. If we had instead chosen to normalize the test-field peaks to 540 pT s^1/2^, our noise floor estimates would improve by ~7% to ~0.84 pT s^1/2^.

## APPENDIX I: FERRITE MICROWAVE FIELD ENHANCEMENT

A feature of our magnetometer is that it uses a simple, nonresonant coil for microwave excitation and only requires 20 mW of microwave power. This is partially enabled by an enhancement of the microwave magnetic field provided by the ferrite cones. Figure 13 shows Rabi oscillations of the same diamond-coil configuration with and without ferrite cones. The Rabi frequency with ferrite is ≿ two times larger, indicating an equivalent ≿ twofold enhancement in the microwave magnetic field.

## APPENDIX J: CALIBRATION OF HELMHOLTZ COILS

Our magnetometer signal’s accuracy relies on a careful calibration of the conversion between the current applied to the Helmholtz coils and *B*_ext_. Here we call this conversion factor *M*_cal_. Theoretically, we estimated *M*_cal_ = 165 μT/A based on the known coil geometry and number of turns. We verified this estimate experimentally by applying currents to the Helmholtz coils and measuring the resulting magnetic field using three different magnetometers.

First, two commercial vector magnetometers (Twinleaf VMR and SENSYS fluxgate; see Fig. 5) were used to calibrate the Helmholtz coils. Each magnetometer was placed in the center of the coils at approximately the same location as the NV-ferrite structure would be. The current in the Helmholtz coils was varied and the axial magnetic field component was recorded. Figure 14(a) shows the resulting calibration curves. The data were fit to linear functions, revealing *M*_cal_ (listed in the legend). For the fluxgate magnetometer, *M*_cal_ is approximately the same as the theoretical estimate when the top of the magnetic shields was removed. When the shield remained in place, the calibration factor was ~5% larger. The VMR magnetometer reported a lower magnetic field than other methods. In both cases we relied on conversion constants between voltage and magnetic field units as provided by the manufacturers.

Note that when the current was turned off, we still observed a small residual axial field of *B*_ext_ = −0.2 μT using both magnetometers. This is due to the finite attenuation provided by the shields. When the shields were removed, the axial component of the laboratory field was approximately −20 μT. Since the shields provide a ~100-fold attenuation, this leads to a small residual field of −0.2 μT.

Next, we used NV magnetometry, with the ferrite cones removed from the setup (Appendix H), to measure the ODMR frequencies as a function of the current in the coils. Figure 14(b) shows the observed *f*_±_ values alongside a fit according to the NV spin Hamiltonian, Eq. (A2), with *M*_cal_ = 175 μT/A as a fitting parameter.

The value of *M*_cal_ used throughout the main text was the average of all three values reported by the magnetometers with the shields on. It is *M*_cal_ = 167 ± 14 μT/A, where the uncertainty is the standard deviation. If we had removed the VMR magnetometer from the analysis, we would have obtained *M*_cal_ ≈ 175 μT/A. This would decrease the reported sensitivity by ~5% to ~0.95 pT s^1/2^.

## APPENDIX K: SENSITIVITY WITHOUT FERRITE

We used the same dual-resonance magnetometry technique described in the main text to record the diamond magnetometry signal with the ferrite cones removed. Figure 15 shows the resulting magnetic noise spectrum alongside the spectrum with ferrite [reproduced from Fig. 4(b)]. The noise floor without ferrite is ~300 pT s^1/2^. This is slightly larger than the expected 254-fold increase, most likely due to a suboptimal choice of microwave power.

## APPENDIX L: FLUX CONCENTRATOR HYSTERESIS

The data in Fig. 2(c) were obtained by sweeping *B*_ext_ from zero to +50 μT, then from +50 μT to −50 μT, and finally from −50 μT back to zero. To check whether hysteresis results in any remanent fields over the course of these measurements, we separated the *f*_±_ data into three segments: 0– + 50 μT, +50− − 50 μT, and −50–0 μT. We fit the three data sets separately according to Eq. (A2) with a residual magnetic field offset of *B*_ext_ as the only fitting parameter. The resulting offset magnetic fields were found to be 8.8 nT, −9.2 nT, and 9.8 nT, respectively. This variation lies within the fit uncertainty, so we take 10 nT as an upper bound. Note that this corresponds to a remanent field within the gap of ≾2.5 μT.

## APPENDIX M: PHOTOELECTRON SHOT-NOISE LIMIT

The photoelectron-shot-noise-limited sensitivity of our magnetometer is given by

(M1) $$\eta_{\text{psn}}=\frac{\sqrt{qI_{\text{dc}}}}{dI_{\text{ac}}/dB_{\text{ext}}},$$

where *I*_dc_ = 2.3 mA is the sum of the average photocurrent in both channels of the balanced photodetector, *dI*_ac_/*dB*_ext_ = 33 Arms/T is the lock-in slope expressed in terms of the AC photocurrent rms amplitude [Fig. 3(c)], and *q* = 1.6 × 10^−19^ C is the electron charge. Thus Eq. (M1) evaluates to *η*_psn_ = 0.58 pT s^1/2^. This noise can be thought of as the standard deviation of the time-domain magnetometer data obtained in 1-s intervals. In the frequency domain it corresponds to the standard deviation of the real part of the Fourier transform expressed in pT s^1/2^. In our experiments, we report the absolute value of the Fourier transform. In order to represent *η*_psn_ in this way, it must be multiplied by 1.25 to reveal a magnetic noise floor of *η*_psn_ = 0.72 pT s^1/2^. This conversion was checked by simulating Poissonian noise and observing the noise floor in the absolute value of the Fourier transform.

A similar value *η*_psn_ ≈ 0.75 pT s^1/2^ was obtained by in serting experimental values from ODMR spectra into Eq. (1). In this case, we used *ξ* = 0.01, *P*_opt_ = 200 mW, Γ = 9 MHz, and *C* = 0.04. The effect of flux concentrators is incorporated by multiplying *γ*_nv_ by *ϵ*. Note that the expression in Eq. (1) refers to the sensitivity to the magnetic field component along the NV axis. Since we use this measurement to infer the total field amplitude (which is directed at 55° with respect to the NV axes), the right-hand side of Eq. (1) must be multiplied by $1/\text{cos}55^{{}^{\circ}}=\sqrt{3}$.

In Sec. I we claimed that the lowest value of Γ/*C* [11] was 1 MHz/0.04. To be accurate, the reported contrast in this paper was 0.05 and the linewidth was 1 MHz. However this experiment measured the projection of the field onto NV axes that were aligned at 35° with respect to the field (the field was aligned normal to a [110]-cut diamond face). Incorporating the projection factor (cos 35° = 0.82) in Eq. (1) has the same effect as scaling down the ratio Γ/*C* by the same proportion. We thus reported the ratio as Γ/*C* ≈ 1 MHz/0.04.

Finally, we would like to clarify some issues with the optimistic estimation of *η*_psn_ made in Sec. I. There, we combined the highest-reported value of *ξ* with the lowest reported value of Γ/*C*. In reality such a combination may be difficult to achieve as there are competing factors. For example, realizing high *ξ* requires high optical depth. This is challenging to realize when is small, because the latter implies a low NV density. In principle this could still be accomplished with a multipass configuration or by using a large diamond. However, as one moves to lower Γ, the optimal excitation intensity also decreases (since the optical excitation rate should not exceed Γ). This means that, for a fixed power, the beam area must increase, which further constrains the geometry and favors larger diamond dimensions. A lower excitation intensity also results in a smaller magnetometer bandwidth, since the optical repolarization rate is lower. Finally, realizing a high value of *ξ* requires getting waveguides and/or lenses very close to the diamond. Realizing such a high optical access may interfere with other magnetometer components (concentrators, microwave loop, heat sinks, etc.). Most of these technical challenges are not insurmountable, but they need to be addressed. Our flux concentrator solution offers a complementary path that may alleviate some of these engineering constraints.

## APPENDIX N: MAGNETOMETER SENSITIVITY UNITS

In this work, we report magnetic sensitivity with the units T s^1/2^. However other magnetometry works report the sensitivity in units of T Hz^−1/2^. This includes the manufacturer-specified sensitivities for the commercial magnetometers we studied in Fig. 5. We justify our choice of units as follows. The theoretical sensitivity equation [Eq. (1)] results in a quantity *η*_psn_ which has units of T s^1/2^ in SI units. These units tell us how the minimum detectable magnetic field scales with integration time. Alternatively, they tell us how the minimum detectable magnetic field scales with sample rate, assuming continuous sampling. In order to convert to T Hz^−1/2^, one has to make an assumption about how the sampling rate converts to measurement bandwidth. If one assumes that only positive frequencies are considered, then, by the Nyquist theorem, a 1 Hz measurement bandwidth would imply a sampling rate of 2/s. Or in other words, Hz^−1/2^ ↔ (0.5 s)^1/2^. This is a common convention used in many metrology communities and in some parts of the NV sensing works [23,53]. However if negative frequencies are considered then a 1 Hz measurement bandwidth could span symmetrically from negative to positive frequencies. In this case, Hz^−1/2^ ↔ s^1/2^. Given this ambiguity, we decided to report the sensitivity in units of T s^1/2^. To analyze the experimental data, we digitized the magnetometer data in the time domain and then processed the Fourier transform digitally. We normalized the Fourier transform in units of T s^1/2^ to remain consistent with the theoretical sensitivity analysis.

## APPENDIX O: MAGNETOMETER ALLAN DEVIATION

As an additional characterization of our magnetometer, we computed the Allan deviation [54] of the magnetometer traces studied in Fig. 4. Figure 16 shows the Allan deviation of the dual-resonance measurement and the case when microwaves were off. Prior to taking the Allan deviation the dual-resonance time-series data were processed with 1-Hz bandwidth notch filters about the test field frequency, the 60 Hz line harmonics up to 960 Hz, and at ten other frequencies in the 7.6–1000 Hz range corresponding to prominent noise peaks. The microwaves-off Allan deviation exhibits *t*^−1/2^ behavior throughout the 100-s data set, as expected for white noise. The dual-resonance Allan deviation approximately follows *t*^−1/2^ scaling up to 0.1 s.

## APPENDIX P: FERRITE THERMAL MAGNETIC NOISE

Thermal magnetic noise originating from dissipative materials can be estimated using fluctuation-dissipation methods [39,49]. The noise is inferred by calculating the power loss (*P*) incurred in the material due to a hypothetical oscillating magnetic field (angular frequency: *ω*) produced by a small current loop (area: *A*, current: *I*) situated at the location of the magnetometer. The magnetic noise detected by the sensor is then given by

(P1) $$\delta B_{\text{gap}}=\frac{\sqrt{8kTP}}{AI\omega },$$

where *k* is the Boltzmann constant. The power loss has separate contributions due to thermal eddy currents and magnetic domain fluctuations:

(P2) $$P_{\text{eddy}}=\int_{V}\frac{1}{2}\sigma E^{2}dV,\;P_{\text{hyst}}=\int_{V}\frac{1}{2}\omega \mu^{\prime \prime }H^{2}dV.$$

Here *σ* is the electrical conductivity, *μ*″ is the imaginary part of the permeability (*μ* = *μ*′ − *iμ*″), *E* and *H* are the amplitudes of the induced electric and magnetic fields, and the integration is carried out over the volume *V* of the dissipative material. In the small excitation limit, *E* and *H* scale linearly with the driving dipole moment (*AI*), so the magnetic noise in Eq. (P1) is independent of the size and driving current in the loop.

We numerically calculated magnetic noise contributions due to *P*_eddy_ and *P*_hyst_ for our flux concentrator geometry [Figs. 1(a) and 1(b)]. We used MN60 material parameters [39] (*σ* = 0.2 Ω^−1^m^−1^, *μ*′= 6500 *μ*_0_, *μ*″= 26 *μ*_0_, where *μ*_0_ is the vacuum permeability) and a cone gap of *δ* = 47 μm, which resulted in the experimental enhancement factor *ϵ* = 254. We find that thermal eddy currents produce white mag netic noise at the level of *δB*_gap_ ≈ 0.02 pT s^1/2^. Since we are interested in our sensitivity in relation to the external field [39], noise produced locally by the ferrite cones translates to an equivalent external field noise of *δB*_ext_ = *δB*_gap_/*ϵ* ≈ 7 × 10^−5^ pT s^1/2^. This negligibly low noise level is a consequence of our choice of low-conductivity ferrite materials. On the other hand, thermal magnetization noise results in a larger, frequency-dependent magnetic noise. At 1 Hz the effective noise is 0.5 pT s^1/2^, and it scales with frequency as *f* ^−1/2^. The thermal magnetization noise is annotated in Fig. 4(b). It is not a limiting factor in our present experiments, but it may have implications for future optimization efforts. If a material with a lower relative loss factor (*μ*″/*μ*′^2^) could be identified, it would result in lower thermal magnetization noise (Appendix Q).

## APPENDIX Q: THERMAL MAGNETIC NOISE FOR VARIOUS MATERIALS

We also used Eqs. (P1) and (P2) to estimate the magnetic noise produced by cones of the same dimensions as in Fig. 1(a), but made from different magnetic materials. Specifically, we considered low-carbon steel 1018 [55], MnZn ferrite MN80 [56], and mu-metal [39]. The results of these estimates are listed in Table I along with the material parameters used for the analysis. In all cases, the hysteresis noise is dominant for frequencies ≾10 Hz.

To minimize hysteresis noise, one must limit the relative loss factor (*μ*″/*μ*′^2^). We found that the hysteresis noise scales proportional to $\sqrt{\mu^{\prime \prime }/\mu^{\prime 2}}$ (Fig. 17). Another design consideration is the geometry of the flux concentrators, but such an optimization is beyond the scope of this work. If the Johnson noise matters, as in the conductive mu-metal, it could be further decreased by passivating the skin effect with a lamination.

Finally, we estimated the magnetic noise produced by the mu-metal magnetic shield used in our experiments. Here, we used an analytical expression for a finite closed cylinder [49] and inserted mu-metal parameters from Table I along with the shield dimensions (height: 150 mm, diameter: 150 mm, thickness: 1.5 mm). The calculated Johnson noise for our shield is *δB*_eddy_ = 0.02 pT s^1/2^ and the hysteresis noise at 1 Hz is *δB*_hyst_ = 0.007 pT s^1/2^. These are much lower than the observed noise floors and can safely be neglected. Note that the noise from the shields is enhanced by the flux concentrators. This effect was incorporated in the calculations, but we still arrived at negligibly low values.

## APPENDIX R: SENSITIVITY TO VARIATION OF THE GAP LENGTH

An important systematic effect in our device could arise from temporal variations in the cone gap length. According to the data in Fig. 1(d), a small change in gap length in the vicinity of *δ* ≈ 43 μm produces a change in the enhancement factor given by *dϵ*/*dδ* ≈ 6/μm. This variation extrapolates to a variation in the magnetometer reading given by

(R1) $$\frac{dB_{\text{ext}}}{d\delta }=\frac{d\epsilon }{d\delta }\frac{B_{\text{ext}}}{\epsilon }.$$

For *ϵ* ≈ 254 and *B*_ext_ = 1.7 μT, Eq. (R1) predicts that a change in *δ* of just 25 pm produces an error in estimation of *B*_ext_ of 1 pT.

Fortunately the gap length remains relatively stable in our construction such that this effect may only be a problem at low frequencies. If the material in the gap expands and contracts due to changes in temperature, this produces a thermal dependence of the magnetometer output given by

(R2) $$\frac{dB_{\text{ext}}}{dT}=\frac{dB_{\text{ext}}}{d\delta }\frac{d\delta }{dT}.$$

If the material in the gap has a thermal expansion coefficient *α*, then the temperature dependence of the gap length is *dδ*/*dT* = *αδ*. For diamond, *α* ≈ 0.7 × 10^−6^ K^−1^. Using this value, and inserting Eq. (R1) into Eq. (R2), we find a magnetometer temperature dependence of *dB*_ext_/*dT* ≈ 1.2 pT/K. For comparison, the temperature dependence due to thermal shifts of *D*(Δ*T*) in single-resonance magnetometry is more than four orders of magnitude larger, $\sqrt{3}/\left(\gamma_{\text{nv}}\epsilon \right)dD/dT\approx -24\text{ nT}/\text{K}$ (Sec. II). Nevertheless, to reach this limit, care must be taken to mechanically stabilize the gap using an approach which does not significantly increase *dδ*/*dT*. For example, using mechanical clamping and/or very thin adhesive layers would be beneficial.

## APPENDIX S: SENSITIVITY TO THERMAL VARIATION OF FERRITE PERMEABILITY

Another possible systematic effect is due to thermal variation of the ferrite relative permeability, *μ*_*r*_. Figure 18(a) plots the expected dependence of MN60 *μ*_*r*_ on *T* as per one manufacturer’s specifications (Ceramic Magnetics, Inc.). Knowledge of *μ*_*r*_(*T*) allows us to simulate the expected thermal variation of the enhancement factor, (*T*), using the finite-element methods described in Appendix B. As seen in Fig. 18(b), the variation in *ϵ*(*T*) is ≾0.2% over the temperature range 275–375 K. This variation is much smaller than the variation in *μ*_*r*_(*T*), but it is not necessarily negligible, particularly for large bias fields.

The right vertical axis of Fig. 18(b) shows the expected shift in magnetometer reading, Δ*B*_ext_(*T*) = *ϵ*(*T*)*B*_ext_/*ϵ*(350 K), as a function of temperature for the bias fields used in our experiments, *B*_ext_ = 1.7 μT. For operating temperatures in the range 305–335 K, the temperature dependence of Δ*B*_ext_(*T*) is ~ − 75 pT/K. This variation is approximately 300 times smaller than that due to thermal shifts of *D*(Δ*T*) in single-resonance magnetometry (Appendix R). However it suggests that the temperature must be stabilized to within ~10 mK in order for this effect to be smaller than the photoelectron-shot-noise-limited sensitivity for frequencies ≾1Hz. Fortunately, for other operating temperatures (~300 K or ~350 K) the first-order variation in *μ*_*r*_ (*T*) vanishes, and thus the dependence of *ϵ* on *T* is minimal. For example, Δ*B*_ext_(*T*) varies by ≾10 pT over the temperature range 348–358 K. Thus, certain bias temperatures may be employed to make the magnetometer more robust against thermal variations.
